# Change in Latent Gray-Matter Structural Integrity Is Associated With Change in Cardiovascular Fitness in Older Adults Who Engage in At-Home Aerobic Exercise

**DOI:** 10.3389/fnhum.2022.852737

**Published:** 2022-05-17

**Authors:** Sarah E. Polk, Maike M. Kleemeyer, Ylva Köhncke, Andreas M. Brandmaier, Nils C. Bodammer, Carola Misgeld, Johanna Porst, Bernd Wolfarth, Simone Kühn, Ulman Lindenberger, Elisabeth Wenger, Sandra Düzel

**Affiliations:** ^1^Center for Lifespan Psychology, Max Planck Institute for Human Development, Berlin, Germany; ^2^International Max Planck Research School on the Life Course (LIFE), Berlin, Germany; ^3^Max Planck UCL Centre for Computational Psychiatry and Ageing Research, Berlin, Germany; ^4^Department of Psychology, MSB Medical School Berlin, Berlin, Germany; ^5^Department of Sports Medicine, Charité – Universitätsmedizin Berlin, Humboldt Universität zu Berlin, Berlin, Germany; ^6^Lise Meitner Group for Environmental Neuroscience, Max Planck Institute for Human Development, Berlin, Germany

**Keywords:** physical activity, fitness, brain structure integrity, aging, older adults, structural equation modeling

## Abstract

In aging humans, aerobic exercise interventions have been found to be associated with more positive or less negative changes in frontal and temporal brain areas, such as the anterior cingulate cortex (ACC) and hippocampus, relative to no-exercise control conditions. However, individual measures such as gray-matter (GM) probability may afford less reliable and valid conclusions about maintenance or losses in structural brain integrity than a latent construct based on multiple indicators. Here, we established a latent factor of GM structural integrity based on GM probability assessed by voxel-based morphometry, magnetization transfer saturation, and mean diffusivity. Based on this latent factor, we investigated changes in structural brain integrity during a six-month exercise intervention in brain regions previously reported in studies using volumetric approaches. Seventy-five healthy, previously sedentary older adults aged 63–76 years completed an at-home intervention study in either an exercise group (EG; *n* = 40) or in an active control group (ACG; *n* = 35). Measures of peak oxygen uptake (VO_2_peak) taken before and after the intervention revealed a time-by-group interaction, with positive average change in the EG and no reliable mean change in the ACG. Significant group differences in structural brain integrity changes were observed in the right and left ACC, right posterior cingulate cortex (PCC), and left juxtapositional lobule cortex (JLC). In all instances, average changes in the EG did not differ reliably from zero, whereas average changes in the ACG were negative, pointing to maintenance of structural brain integrity in the EG, and to losses in the ACG. Significant individual differences in change were observed for right ACC and left JLC. Following up on these differences, we found that exercising participants with greater fitness gains also showed more positive changes in structural integrity. We discuss the benefits and limitations of a latent-factor approach to changes in structural brain integrity, and conclude that aerobic fitness interventions are likely to contribute to brain maintenance in old age.

## Introduction

As a result of progress in global health and development, the worldwide population of individuals over 60 years old is projected to double by 2050 (HelpAge International, 2018). However, as the proportion of older adults in the population increases, so does the concern of health-related changes associated with advancing adult age. Brain health is of great societal importance, as the risk of cognitive impairments leading to dementia increases with age, and individual differences in the degree to which brain structure, function, and neurochemistry can be maintained into old age are hypothesized to predict individual differences in cognitive functioning among older adults (Nyberg et al., 2012; Lindenberger, 2014; Cabeza et al., 2018; Nyberg and Pudas, 2019; Nyberg and Lindenberger, 2020; Johansson et al., 2022).

Aerobic exercise shows promise as a modifiable lifestyle factor that may potentially promote brain maintenance in old age. Intervention studies focusing on volumetric characteristics of brain structure have found that gray-matter (GM) volume shows more positive changes in exercisers than control participants in frontal areas, such as the anterior cingulate cortex (ACC), and in temporal areas, such as the hippocampus (Colcombe et al., 2004; Erickson et al., 2011). Higher levels of and more positive changes in physical fitness have also been associated with greater GM volume and attenuation of volume loss in prefrontal, parietal, and temporal regions, including the hippocampus (Colcombe et al., 2003; Weinstein et al., 2012; Maass et al., 2015; Kleemeyer et al., 2016).

However, individual measures such as GM probability may afford less valid conclusions about maintenance or losses in structural brain integrity on a generalized level than a latent construct based on multiple indicators. Latent constructs express the variance shared by multiple measures, thereby separating common variance from specific variance and measurement error (Wansbeek and Meijer, 2001). Therefore, the use of latent factors can improve the estimation of associations among constructs of interest. Based on pioneering work by Kühn et al. (2017), Köhncke et al. (2021) introduced a multi-trait multi-method model using structural equation modeling (SEM) capturing the shared variance between GM volume, mean diffusivity (MD), and magnetization transfer (MT) ratio in a latent factor of GM structural integrity for several regions of the brain. The authors were able to show that, in a cross-sectional sample, older participants generally showed lower scores on these integrity factors, which they interpreted as a reflection of age-related deterioration of overall GM, in line with studies focusing on single indicators (Raz et al., 2005; Fjell and Walhovd, 2010; Grydeland et al., 2013; Seiler et al., 2014). In addition, GM structural integrity correlated positively with episodic memory performance (Köhncke et al., 2021).

In the current intervention study, we adapted this cross-sectional model of GM structural integrity to a longitudinal context in order to measure change in structural integrity as a latent factor in brain regions of interest previously reported in studies using volumetric approaches. The three indicators used in the current model were all measured using magnetic resonance imaging (MRI) and captured different characteristics of GM structural integrity. GM volume was calculated using T_1_-weighted images and voxel-based morphometry (VBM; Ashburner and Friston, 2000; Good et al., 2001), which estimates GM concentrations at each voxel based on signal intensity. MT saturation, an improvement to the MT ratio measure, which is affected by spatial variations of the transmit field for excitation and the local T_1_ relaxation (Helms et al., 2008b), was used in the current models. MT maps quantify the transfer of magnetization between tissue water and protons bound to macromolecules, and can be used to assess microstructural changes to GM, with lower MT values correlating with demyelination and axonal loss (see Seiler et al., 2014). MD, measured using diffusion-weighted imaging (DWI), estimates the rate of water diffusion in each voxel (Pierpaoli and Basser, 1996), and is commonly used as a measure of white matter integrity, but can also be used as an index of GM density, with greater MD values corresponding to lower tissue density, likely reflecting demyelination (Song et al., 2005) and lower axon fiber density (Beaulieu, 2002; Fukutomi et al., 2019). By combining these three imaging modalities, we established a latent factor of GM structural integrity representing the commonalities across these three measures of brain structure. We use this novel longitudinal modeling approach to look at exercise-induced changes in a latent factor that represents general structural integrity, focusing on the shared variance of GM volume, MT, and MD, rather than any one of these alone, thereby removing any modality-specific measurement error. Given this emphasis on the commonalities across the individual modalities and to acknowledge the level of abstractness of this latent factor, we refer to this factor as “integrity.”

### The AKTIV Study

Previous studies investigating the effects of exercise among older adults generally have been conducted in a laboratory setting (e.g., Colcombe et al., 2006; Erickson et al., 2011), which may not reflect the exercise opportunities accessible to most older adults in their everyday lives. To examine whether engaging in aerobic exercise at home may also benefit older adults in terms of both fitness and brain health, the “Aktives Altern für Körper und Geist” (active aging for body and mind) study (AKTIV) implemented a personalized at-home physical exercise regime.

In the current analyses, we first investigated whether six months of moderate, at-home aerobic exercise could effectively boost cardiovascular fitness in older adults. We then validated the GM structural integrity model in a longitudinal manner in 12 regions of interest (ROIs) in the frontal, midline, and temporal areas selected based on previous publications (Colcombe et al., 2006; Erickson et al., 2011), and examined whether the group who exercised showed either gains (increase over controls) or maintenance (attenuation of loss relative to controls) in GM structural integrity within these regions. Finally, we investigated the association between changes in cardiovascular fitness and changes in structural brain integrity, with the hypothesis that greater increases in fitness should be positively associated with greater increases (or smaller losses) in GM structural integrity.

## Materials and Methods

### Sample and Study Design

In the current analyses, we focused on the effects of physical training on GM structural integrity by comparing a group of individuals who regularly engaged in aerobic exercise with a group of sedentary individuals. These two groups were drawn from the larger AKTIV study, which investigated the effects of cognitive and physical training in comparison to an active control group (ACG). Here, we explored the effects of physical training alone vs. no physical training (see Interventions for details) with a sub-sample of 75 healthy, previously sedentary adults aged 63–76 years.

A full description of the AKTIV study design and methods can be found in Wenger et al. (2022); for convenience, we describe relevant materials and methods here. Volunteers for the AKTIV study were recruited through a participant data bank with participants from earlier, unrelated studies, and newspaper advertisements. A telephone screening was conducted to exclude individuals if they met any of the following criteria: MRI contraindications; inability to meet the time requirements of the study; not right-handed; younger than 63 or older than 78 years old at the start of the study; already engaging in aerobic exercise more than once every 2 weeks; fluent in a language other than German or English, or fluent in more than two languages; receiving medical treatment for Parkinson’s, gout, rheumatism, heart attack, stroke, cancer, severe back problems, severe arrhythmia, severe chronic liver or kidney failure, severe disease of the hematopoietic system, mental illness (e.g., depression), or neurological disease (e.g., epilepsy, brain tumor). The 201 volunteers who did not meet the exclusion criteria were randomly assigned (with the exception of couples who were jointly assigned so that participants would remain blind to other groups) to one of four groups: (1) an ACG, (2) a language training group, (3) a physical exercise training group, or (4) a combined language and physical exercise training group. Next, potential participants were invited to a physical assessment including cardiopulmonary exercise testing (CPET) with lactate diagnostics at the Department of Sports Medicine at the Charité – Berlin University of Medicine. Based on this exam, a further 22 volunteers dropped out or were excluded due to existing medical conditions. Finally, participants underwent an initial MRI session before beginning their assigned training [pre-intervention, time point 1 (T1)]. Nineteen participants dropped out before the training started due to disinterest and one additional participant was excluded due to claustrophobia. Thus, the effective initial sample consisted of 159 individuals.

After 3 months of training [mid-intervention, time point 2 (T2)], MRI acquisitions were repeated, consisting of the same scans. After a total of 6 months of training [post-intervention, time point 3 (T3)], MR measures were acquired once more, and participants again underwent CPET at the Charité.

During the intervention, 17 participants dropped out due to physical complaints (e.g., knee or back pain during training), disinterest, time constraints, or unspecified reasons.

The Ethics Committee of the German Psychological Society (DGPs) approved the study and written informed consent was collected from all participants.

### Interventions

As previously stated, we focused on the effects of physical exercise and therefore only used the data of participants who completed the intervention in the exercise-only group or the ACG.

Participants in the exercise group (EG; *n* = 40 completed, mean age = 69.8 years, 50% females) engaged in moderate at-home exercise three to four times per week at any time of the day using a bicycle ergometer (DKN Ergometer AM-50) and a personalized interval training regime programmed onto tablets (Lenovo TB2-X30L TAB) that were synced to the ergometers *via* Bluetooth. Tablets were equipped with SIM cards so that data could be uploaded to the study server whenever an Internet connection was available. The training initially lasted 30 min at an individually set intensity (25–140 W, mean = 67.9, SD = 26.65). After completing each training session, participants could indicate if they found the training too easy or too difficult, and the intensity could be remotely adjusted accordingly. In this way, the training was highly personalized so that participants would not be discouraged by an exceedingly easy/difficult exercise program. Approximately every two weeks, difficulty was automatically increased by 3 min per interval (up to 56 min total) and 3–4 W. Participants in the EG were also instructed to read pre-selected literature on the tablet at a slow pace for an additional 15 min on days when they trained and for 45 min on the other days, so that participants in both groups would engage in approximately 45 min of study-related activity on at least six days per week. Finally, those in the EG participated in weekly 1-h group sessions (5–10 participants per session) at the institute consisting of toning and stretching, led by an external instructor.

Participants in the ACG (*n* = 35 completed, mean age = 70.7 years, 40% females) also received a tablet and were instructed to read pre-selected literature for 45 min daily for at least six days per week. These participants also attended weekly group sessions during which they discussed literary excerpts led by external facilitators.^1^

Compliance was defined as engaging in at least an average of 90 min of group-relevant activity (reading or exercise) per week over 21 weeks (≥1890 min; *n*_*non*–*compliant*_ in ACG = 3, *n*_*non–compliant*_ in EG = 4) with no pauses of longer than 2 weeks (*n*_*non–compliant*_ in EG = 1). Participants in the EG also needed to exercise at a steady or slightly increasing intensity (based on Watts) over the duration of the intervention, meaning those with decreasing Wattage were counted as non-compliant (*n*_*non–compliant*_ in EG = 5).

Finally, regarding sample size, *post hoc* sensitivity analyses conducted in G*Power (version 3.1.9.6; Faul et al., 2007, 2009) indicated that, with α = 0.05, 1 − β = 0.95, and a study design with two groups and three time points, a sample size of *N* = 75 could reliably capture time-by-group interaction effects with an effect size of *f* ≥ 0.19 and correlations with a coefficient of *r* ≥ 0.367.

### Data Acquisition

#### Cardiovascular Fitness

Cardiovascular fitness, indexed by peak oxygen uptake (VO_2_peak; measured at 30-s intervals, relativized to body weight in kg), was assessed at the physical assessments at pre- and post-intervention using CPET. This was conducted under the supervision of the overseeing physician using a bicycle ergometer (Ergoselect 100k, Ergoline GmbH, Bitz, Germany) using the Quark Clinical-based Metabolic Cart with the standard Breath-by-Breath setup, and the V2 Mask (Hans Rudolph, Inc.), which covers the mouth and nose, and is fastened to the back of the head. Participants were instructed to pedal at a constant rate of 60–70 rotations per minute during the entire protocol, which consisted of a 3-min rest phase, an exertion phase with a starting resistance of 20 W, which increased by 20 W every 3 min until participants reported they had reached maximum exertion, and a 5-min recovery phase with no resistance.

#### Magnetic Resonance Imaging

##### Acquisition

Participants were scanned pre-, mid-, and post-intervention. MR images were acquired using a 3T Magnetom Tim Trio MRI scanner system (Siemens Medical Systems, Erlangen) using a 32-channel radiofrequency head coil. T_1_-weighted images were obtained using a 3D T_1_-weighted magnetization prepared gradient-echo (MPRAGE) sequence. The multi-parameter mapping protocol used to acquire the MT maps comprised one static magnetic (B_0_) gradient echo (GRE)-field map, one radiofrequency (RF) transmit field map (B_1_), and three multi-echo 3D fast low angle shot (FLASH) scans (Helms et al., 2008b; Tabelow et al., 2019). Diffusion-weighted images were obtained with a single-shot diffusion-weighted spin-echo-refocused echo-planar imaging sequence. Further details regarding the acquisition parameters can be found in the Supplementary Material.

##### Preprocessing

Structural T_1_-weighted images were preprocessed using the Computational Anatomy Toolbox 12 (CAT12, Structural Brain Mapping group, Jena University Hospital; Gaser and Dahnke, 2016) in Statistical Parametric Mapping (SPM12, Institute of Neurology^2^) using the default parameters of the longitudinal pipeline. Estimation of MT maps was conducted in SPM12 using an adapted longitudinal pipeline with the hMRI toolbox (Tabelow et al., 2019^3^). DW images were preprocessed using MRtrix (version 3.0_RC3; Tournier et al., 2019), FSL (FMRIB’s Software Library, version 6.0.2; Smith et al., 2004; Woolrich et al., 2009; Jenkinson et al., 2012), and ANTS (version 2.2.0; Avants et al., 2010, 2011), following the Basic and Advanced Tractography with MRtrix for All Neurophiles (B.A.T.M.A.N.) tutorial (Tahedl, 2018). Further details regarding preprocessing can be found in the Supplementary Material.

##### Regions of Interest

Regions of interest were selected based on previous intervention studies on the effects of aerobic exercise on brain structure (Colcombe et al., 2006; Erickson et al., 2011). Mean values of VBM, MT, and MD were extracted from the left and right hippocampus, ACC, posterior cingulate cortex (PCC), precentral gyrus (PCG), juxtapositional lobule cortex [JLC, previously supplementary motor cortex (SMA)], and pars triangularis and pars opercularis combined as inferior frontal gyrus (IFG), as defined by the Harvard--Oxford cortical and subcortical structural atlases^4^,^5^ (Desikan et al., 2006), for a total of 12 ROIs. VBM and MT maps were calculated such that each participant’s resulting map was in MNI space, so ROI masks were simply resized using the *Coregister and reslice* module in SPM12 with the first participant’s map at T1 as a reference, and *fslstats* in FSL was used to extract the mean and SD across all non-zero voxels within each ROI. As calculation of MD maps resulted in images in each participant’s native space, ROI masks were first transformed into native space for each participant using *applywarp* in FSL with the transformations generated during preprocessing and used to transform the non-diffusion-weighted images into native space, then *fslstats* was used to extract means and SDs across all non-zero voxels.

VBM values from each ROI were adjusted for intracranial volume [ICV, calculated using the *Estimate TIV and global tissue volumes* module in CAT12] using the analysis of covariance formula from Raz et al. (2005): adjusted volume = raw volume − *b* × (ICV − mean ICV), where *b* is the slope of regression of GM probability in the relevant ROI on ICV.

### Statistical Analyses

All statistical analyses were conducted using R (R Core Team, 2021), version 4.1.2 (2021-11-01), in RStudio (RStudio Team, 2021), version 2021.09.1.

#### Univariate and Multivariate Outlier Detection

Univariate outliers within each measure and time point were defined as data points further than 4 SD away from the mean, resulting in one data point being discarded (hippocampus MT at T3). Multivariate outlier detection was conducted within measures across all three time points (two time points for VO_2_peak) for complete cases using *robustMD* from the faoutlier R package (Chalmers and Flora, 2015) with the classical product-moment method (criterion = 0.001). In this way, we looked for abnormal patterns across time points within our sample (e.g., one data point having a much higher value than the other two), and removed all three data points of such outliers. This resulted in no cases being discarded for VO_2_peak, and 17 out of 2052 cases being discarded across the three MRI modalities and 12 ROIs in participants with all three observations.

#### Structural Equation Modeling

SEM was used to investigate differences in group means of change in cardiovascular fitness indexed by VO_2_peak and latent GM integrity, as well as the relationship between these two changes using a multivariate, multigroup approach. Models were specified and estimated using the OpenMx R package (Pritikin et al., 2015; Neale et al., 2016; Hunter, 2018; Boker et al., 2021), version 2.19.8. Full information maximum likelihood (FIML) was used to account for missing data without the need for case-wise deletion. Observed variables were rescaled to have a mean of 0 and a SD of 1 longitudinally (i.e., data from all time points were first stacked then rescaled) to preserve the relative mean differences between the same indicators measured at different time points. The root mean square error of approximation (RMSEA) and the comparative fit index (CFI) were used to evaluate model fit, using rough thresholds of RMSEA < 0.08 and CFI > 0.90 to indicate acceptable model fit (Schermelleh-Engel et al., 2003).

Likelihood-ratio tests (LRTs) were used to determine the statistical significance of group differences on individual parameters, as well as of certain parameter estimates within a model. To conduct an LRT, a model in which the parameter(s) of interest is freely estimated is compared to a nested model in which this parameter is fixed (e.g., to 0 or equal across groups). The difference in χ^2^ (i.e., the likelihood ratio) between the two models indicates the difference in fit, and if this difference is significant, the null hypothesis that the models fit equally well can be rejected (Kline, 2016). Given previous studies on the effects of aging and exercise on brain volume and structural integrity (Colcombe et al., 2006; Erickson et al., 2011; Köhncke et al., 2021), we had strong hypotheses that exercise would be beneficial to structural integrity of the brain. That is, those participants who exercised should show gains in or maintenance of structural integrity, while those who did not exercise would show declines in integrity. Therefore, unless otherwise indicated, one-sided hypothesis testing was conducted looking for changes in the positive direction within the EG and in the negative direction within the ACG.

**FIGURE 1:**
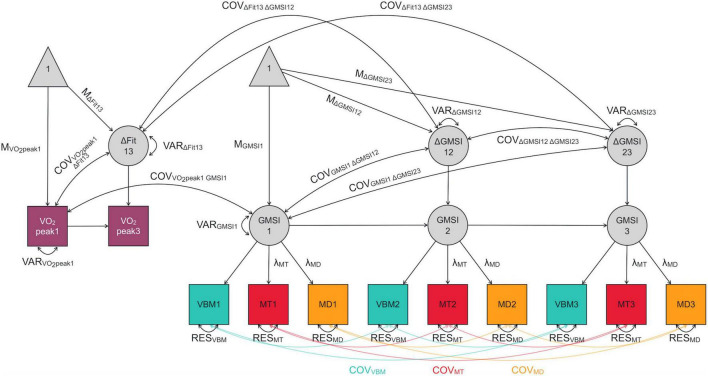
Exemplary bivariate latent change score model measuring the covariance between change in cardiovascular fitness from T1 to T3 and change in gray-matter structural integrity in a given region of interest from T1 to T2 and T2 to T3. Fit, cardiovascular fitness; GMSI, gray-matter structural integrity; VBM, voxel-based morphometry-derived gray-matter probability; MD, mean diffusivity; MT, magnetization transfer saturation; 1, time point 1 (T1; 0 months); 2, time point 2 (T2; 3 months); 3, time point 3 (T3; 6 months); Δ, change; M, mean; COV, covariance; VAR, variance; λ, loading; RES, residual. Covariances between manifest variables of integrity were set to be equal within each modality, denoted by color-coding; these are only labeled once for visual clarity. Other unlabeled paths are fixed to 1.

**FIGURE 2:**
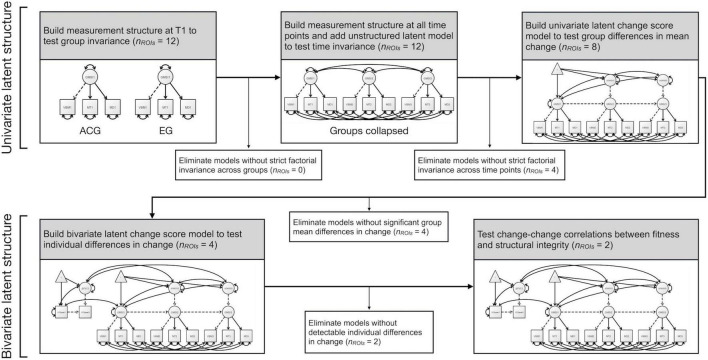
Flowchart of the analysis plan of gray-matter structural integrity models including factorial invariance testing across groups and time points, testing for group mean differences in change, testing for significant individual differences in change, and testing for change-change correlations with fitness. T1, time point 1 (0 months). Model paths intentionally unlabeled for visual clarity. Dashed lines represent paths fixed to 1. See Figure 1 for further model details.

Latent change score models (LCSMs) were used to evaluate group mean differences in change in VO_2_peak and GM integrity following the tutorial by Kievit et al. (2018). Full details of the model validation can be found in the Supplementary Material, but we describe the models briefly in the following. A univariate LCSM was built to measure mean change in VO_2_peak; a pseudo-latent factor, ΔVO_2_peak, captured the difference in VO_2_peak between T1 and T3. Multigroup models were used to test for differences in change between the two groups in cardiovascular fitness. Multivariate LCSMs with three time points were built to measure mean change in GM structural integrity from T1 to T2 as well as from T2 to T3 in each ROI individually. As is common practice in SEM, factorial invariance testing was performed to establish whether the measurement structure of the structural integrity models held across groups and time points. In those regions that survived invariance testing, mean differences in change between the two groups were investigated. Finally, in those models showing detectable individual differences in change as well as significant mean differences in change between groups, change-change correlations between fitness and GM structural integrity were examined using bivariate LCSMs (see Figure 1) both in the whole sample as well as separately for the two groups. This strategy was chosen to add credibility to a causal interpretation of change-change associations in the exercise group (see Ghisletta and Lindenberger, 2004). For an overview of the analysis plan of GM structural integrity models, see Figure 2.

## Results

A description of the sample can be found in Table 1. Participants who did not meet compliance criteria were excluded from T2 and T3 (*n*_*ACG*_ = 3, *n*_*EG*_ = 10) but were kept in at T1 under the assumption that they did not differ from other participants at baseline. One further exercise participant was excluded from T2 and T3 due to technical difficulties.

**TABLE 1:** Sample demographics and intervention specifics.

	Active control group	Exercise group
*n* completed intervention	35	40
*n* fully complied	32	29
Age at baseline, M/SD (range)	70.7/3.81 (64.0–76.0)	69.8/3.49 (63.9–76.9)
Sex, % of female participants	40.0	50.0
Years of education, M/SD (range)	13.4/3.27 (7–16)	13.2/3.02 (7–16)
DSST score at baseline, M/SD (range)	47.3/9.42 (31–68)	45.2/10.90 (21–75)
Total minutes spent in intervention, M/SD (range)	4772/1819.6 (2505–10,858)	6554/1222.9 (4098–9790)
Minutes spent reading, M/SD (range)	4772/1819.6 (2505–10,858)	3381/1143.3 (915–5855)
Minutes spent exercising, M/SD (range)	–	3173/410.4 (2556–3937)

*M, mean; SD, standard deviation; DSST, Digit Symbol Substitution Test. Ms and SDs of age at baseline, sex, years of education, and DSST score at baseline are calculated within participants who completed the intervention. Total minutes spent in intervention are calculated within participants who fully complied to the intervention.*

### Cardiovascular Fitness

**FIGURE 3:**
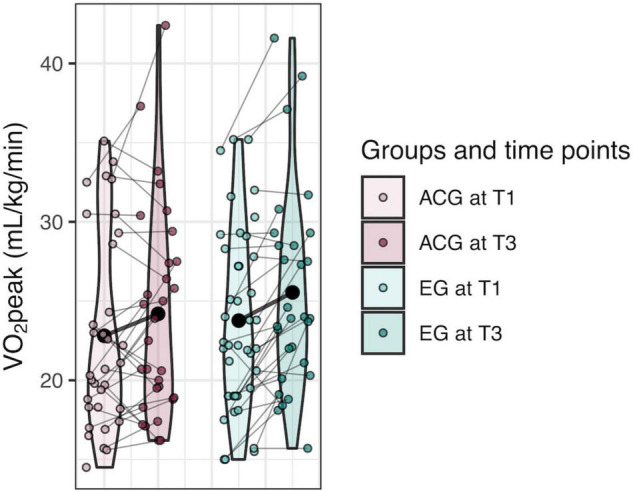
Violin plots of VO_2_peak showing individual trajectories as well as mean change of cardiovascular fitness change over six months by group. T1, time point 1 (0 months); T3, time point 3 (6 months). Large black point represents group mean at each time point.

**FIGURE 4:**
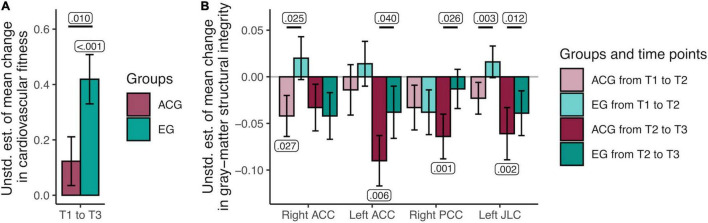
Unstandardized parameter estimates of mean changes in cardiovascular fitness and gray-matter structural integrity by group. **(A)** Results of the univariate latent change score model (LCSM) of VO_2_peak indicated that the active control group showed no mean changes in fitness, whereas the exercise group showed a significant mean increase in fitness over time. **(B)** Results of the multivariate LCSM of gray-matter structural integrity indicated that the active control group showed significant mean decreases in latent gray-matter structural integrity over time in the left and right anterior cingulate cortex, right posterior cingulate cortex, and left juxtapositional lobule cortex (previously supplementary motor area), while the exercise group exhibited maintenance in integrity in these regions. Unstd. est., unstandardized estimate; T1, time point 1 (0 months); T2, time point 2 (3 months); T3, time point 3 (6 months); ACG, active control group; EG, exercise group. Significant *p*-values of one-sided *t*-tests of individual parameters against zero (negative in ACG and positive in EG) as well as differences in group mean change are displayed. Error bars represent estimated SEs.

A univariate LCSM with one pseudo-latent difference score is exactly identified, therefore fit indices are perfect by definition. Cardiovascular fitness as indexed by VO_2_peak showed a significantly positive mean change in the EG, unstandardized estimate of the mean (*b*_0_) = 0.419 (i.e., average in increase of 0.419 mL/kg/min from T1 to T3), standard error (SE) = 0.089, Δχ^2^_(_*_*df*_*_=__1)_ = 18.67, *p* < 0.001, whereas mean change in the ACG was not significant, *b*_0_ = 0.123, SE = 0.088, Δχ^2^_(_*_*df*_*_=__1)_ = 1.92, *p* > 0.050. Furthermore, the EG showed significantly greater mean change than the ACG, Δχ^2^_(_*_*df*_*_=__1)_ = 5.37, *p* = 0.010 (see Figures 3, 4). In terms of percent change, the EG showed a mean change of 12.8% (SE = 2.28), while the ACG showed a mean change of 3.7% (SE = 2.22). A significant difference in VO_2_peak between males and females was seen at baseline, *t*(70.3) = 3.95, *p* < 0.001, with males showing higher VO_2_peak (mean = 25.5, SD = 5.99) than females (*M* = 20.7, SD = 4.46). Males and females did not differ in percent change in VO_2_peak, and no associations of age or years of education were seen with either baseline or percent change in VO_2_peak.

### Gray-Matter Structural Integrity

Out of the 12 initial GM structural integrity models, eight survived testing for factorial invariance, which implies that the factor structure did not vary across groups or time points: right and left hippocampus, right and left ACC, right PCC, right and left JLC, and right IFG. For these models, all standardized factor loadings were significant (*p*s < 0.050). In general, the variable with the highest loading was MD (average standardized loading = −0.857), revealing that this measure was the strongest indicator of latent GM structural integrity; next was VBM (0.527), followed by MT (0.463). See full model set-up results in Table 2 for more details. Results for combined left and right hemispheres can be found in Supplementary Material.

**TABLE 2:** Testing for invariance across groups and time points, and testing for equal vs. unequal mean change parameters across groups.

	Group invariance (T1)		Time invariance (groups collapsed)		Standardized estimates of factor loadings			Group difference in mean change	
Region of interest	Δχ^2^_*df*__=__2_ baseline vs. metric	Δχ^2^_*df*__=__3_ metric vs. strict	Δχ^2^_*df*__=__4_ baseline vs. metric	Δχ^2^_*df*__=__6_ metric vs. strict	VBM	MT	MD	Δχ^2^_*df*__=__1_ ΔSI T1 to T2	Δχ^2^_*df*__=__1_ ΔSI T2 to T3
Right HC*	0.33	4.34	0.69	4.67	0.792^†^	0.576^†^	−0.887^†^	0.48	0.09
Left HC*	0.51	4.19	2.18	4.51	0.751^†^	0.464^†^	−0.987^†^	1.33	0.42
Right ACC*	2.82	2.49	6.08	2.53	0.596^†^	0.518^†^	−0.895^†^	3.87^†^	0.38
Left ACC*	1.78	1.20	5.60	8.06	0.278^†^	0.632^†^	−0.256^†^	1.85	3.07^†^
Right PCC*	4.01	0.69	0.00	4.49	0.494^†^	0.418^†^	−1.000^†^	0.75	3.75^†^
Left PCC	0.00	6.21	17.12^†^	–	–	–	–	–	–
Right PCG	0.00	1.28	10.16^†^	–	–	–	–	–	–
Left PCG	4.64	2.33	10.85^†^	–	–	–	–	–	–
Right JLC*	1.57	3.29	1.07	12.19	0.412^†^	0.417^†^	−0.942^†^	0.95	1.87
Left JLC*	1.15	0.90	2.82	7.49	0.478^†^	0.415^†^	−0.931^†^	7.45^†^	5.05^†^
Right IFG*	0.44	5.37	0.00	9.77	0.416^†^	0.263^†^	−0.957^†^	2.34	0.15
Left IFG	5.02	1.44	19.10^†^	–	–	–	–	–	–

*HC, hippocampus; ACC, anterior cingulate cortex; PCC, posterior cingulate cortex; PCG, precentral gyrus; JLC, juxtapositional lobule cortex; IFG, inferior frontal gyrus; ΔSI, change in structural integrity.*

*Model shows factorial invariance across groups and time points.

*^†^p < 0.050. Factorial invariance can be assumed if the measurement invariance test is non-significant (two-sided), meaning the model fit does not significantly worsen if parameters are set to be equal across groups/time points. Significance of standardized factor loadings tested using a Wald test (one-sided). A group difference in mean change can be inferred if the p-value is significant (one-sided), meaning the model fit significantly worsens when the parameter is constrained to be equal across groups.*

#### Group Differences in Change

Group differences in mean change in structural integrity from T1 to T2 and from T2 to T3 were tested in those models that showed factorial invariance across groups and time points. All univariate LCSMs had acceptable fit indices, RMSEAs < 0.062 (95% CIs = [0, <0.116]), CFIs > 0.988. The EG showed significantly more positive change than the ACG in the right ACC from T1 to T2, in the left ACC from T2 to T3, in the right PCC from T2 to T3, and in the left JLC from T1 to T2 and from T2 to T3 (see Table 2 and Figure 4 for details). These group differences were primarily driven by mean decreases in GM integrity within the ACG: right ACC from T1 to T2, *b*_0_ = −0.042, SE = 0.022, Δχ^2^_(_*_*df*_*_=__1)_ = 3.68, *p* = 0.027, left ACC from T2 to T3, *b*_0_ = −0.090, SE = 0.027, Δχ^2^_(_*_*df*_*_=__1)_ = 6.44, *p* = 0.006, right PCC from T2 to T3, *b*_0_ = −0.064, SE = 0.024, Δχ^2^_(_*_*df*_*_=__1)_ = 9.05, *p* = 0.001, left JLC from T2 to T3, *b*_0_ = −0.061, SE = 0.028, Δχ^2^_(_*_*df*_*_=__1)_ = 8.73, *p* = 0.002. GM structural integrity did not significantly decline in the left JLC within the ACG from T1 to T2, nor did it significantly increase within the EG in any of the ROIs showing significant differences in group mean change. These time-by-group interaction effects were also detected when including age, sex, and years of education as indicator variables in the models, estimating means and residual variances of each, as well as regressions from each to baseline GM structural integrity, change from T1 to T2 and change from T2 to T3. In the following, we therefore discuss the simpler models, excluding these demographic factors, for the sake of parsimony.

### Correlations Between Change in Cardiovascular Fitness and Change in Gray-Matter Structural Integrity

Two models, the right ACC and the left JLC, showed group differences in mean changes and reliable variance in change, thereby allowing us to investigate whether individual differences in fitness changes and individual differences in integrity changes were correlated. Both models had acceptable fit indices, RMSEAs < 0.059 (95% CIs = [0, <0.106]), CFIs > 0.987. Change in cardiovascular fitness was positively correlated with change in GM structural integrity in the right ACC from T1 to T2, standardized estimate (ϕ) = 0.753, Δχ^2^_(_*_*df*_*_=__1)_ = 11.60, *p* < 0.001, and in the left JLC from T1 to T2, ϕ = 0.469, Δχ^2^_(_*_*df*_*_=__1)_ = 6.42, *p* = 0.006. Crucially, we observed a group difference in change-change correlation in the right ACC from T1 to T2, Δχ^2^_(_*_*df*_*_=__1)_ = 4.52, *p* = 0.017, with the ACG showing no significant correlation, ϕ = 0.424, Δχ^2^_(_*_*df*_*_=__1)_ = 1.92, *p* > 0.050, and the EG showing a significantly positive correlation, ϕ = 1.110, Δχ^2^_(_*_*df*_*_=__1)_ = 10.54, *p* = 0.001. These correlations were also detected when including age, sex, and years of education as indicator variables in the models, thus we discuss the results from the simpler models in the following.

## Discussion

This study used a multivariate, multigroup approach in SEM to investigate the effects of six months of at-home aerobic exercise on cardiovascular fitness and a latent measure of GM structural integrity comprising multiple MR imaging modalities, which may be a more reliable measure of structural integrity than individual MR measures, such as GM volume. Change-change relationships between fitness and GM structural integrity were also explored.

### Cardiovascular Fitness

Participants who engaged in interval training on a stationary bike at home for three to four days a week showed an increase in cardiovascular fitness, indexed by VO_2_peak, over six months, and also improved more than an ACG who did not engage in regular aerobic exercise. This serves as a proof of concept for the current study, showing that the exercise intervention utilized in this sample was effective at improving cardiovascular fitness. Notably, the current design differs from previous exercise interventions that looked at exercise-induced changes in the brain in at least two dimensions: firstly, participants exercised at their own convenience in their homes, only coming to the lab once a week for a group stretching and toning session. This corroborates previous studies showing that regular at-home aerobic exercise can also improve cardiovascular fitness in older adults (King, 1991; Salvetti et al., 2008). This finding is important as regular exercise at home with only one supervised stretching and toning per week may be more accessible for an older population than personal training in a facility multiple times a week.

In addition to training taking place in participants’ homes, the exercise regimes were also highly personalized and flexible. Initial difficulty for each participant was individually determined by a sports medicine physician, and during the intervention, participants could indicate the subjective perception of difficulty (i.e., too easy, too difficult) so that subsequent training could be modified accordingly. Participants were also not supervised during the exercise bouts. Still, this adaptive, at-home interval training regime was effective at increasing the cardiovascular fitness of those in the EG over those in the ACG. This is also in line with research on fitness improvements in aging; for example, one study found that older adults who adhered to a six-month exercise program at home under no supervision had greater VO_2_peak at post-test than those who did not adhere, while the groups did not differ at baseline (Morey et al., 2003). Taken together, an adaptive, at-home aerobic exercise regime seems to be an effective intervention for improving cardiovascular fitness in healthy, previously sedentary older adults.

### Gray-Matter Structural Integrity

The statistical analyses used in this study build on previous work (Kühn et al., 2017; Köhncke et al., 2021) in which a multimodal latent factor measuring GM structural integrity was established in a cross-sectional sample, and expanded this model for use in a longitudinal intervention study including three measurement time points to investigate patterns of change. Our results indicate that exercise promotes the maintenance (i.e., attenuated decrease) of structural integrity in regions of the brain that previously have been found to increase in volume in the course of an exercise intervention (Colcombe et al., 2006), namely the right and left ACC, the right PCC, and the left JLC (also termed supplementary motor area). These areas have been shown to undergo substantial age-related atrophy (e.g., Raz et al., 2005; Fjell et al., 2009), with exaggerated posterior atrophy found in patients with Alzheimer’s disease (Lehmann et al., 2011). However, the vulnerability of brain structure to age-related effects in these regions seems to be accompanied by increased amenability to exercise- and physical fitness-induced maintenance and/or gains in older age (see also Colcombe et al., 2006; Ruscheweyh et al., 2011). It has been suggested before that intra-cortical myelin content may be one potentially important mechanism here (Walhovd et al., 2016), with high-myelin regions being more resistant to change, and regions with lower myelin content, such as the medial temporal lobe and cingulate cortices (Grydeland et al., 2013), being more prone to change in both the negative and positive direction.

The latent factor of GM structural integrity as established in this study captured the variance common to VBM, MT, and MD. Köhncke et al. (2021) reported that older individuals showed lower factor scores in the prefrontal cortex, hippocampus, and parahippocampal gyrus. This suggests that lower factor scores may be indicative of greater GM deterioration that occurs during normal aging, which could be caused by various and potentially correlated structural changes, including loss of dendritic spines and dendritic arbors, decreasing synaptic density, demyelination, and loss of glia and small blood vessels (Hof and Morrison, 2004; Morrison and Baxter, 2012; Zatorre et al., 2012; Raz and Daugherty, 2018). Considering the single indicators, the factor loadings indicate that lower factor scores result from a pattern of lower VBM, lower MT, and higher MD values, which are thought to reflect lower estimates of GM volume, myelination, and density, respectively. Therefore, a factor score capturing the shared variance between these indicators seems to represent general properties of GM structure that decline with age. Further supporting their interpretation that the latent factor reflects structural integrity, Köhncke et al. (2021) were also able to show a positive association between the latent factor and a latent factor comprising four episodic memory tasks, which is in line with the brain maintenance hypothesis that brain integrity across multiple levels is important for cognitive performance (Nyberg et al., 2012; Lindenberger, 2014; Cabeza et al., 2018; Nyberg and Pudas, 2019; Nyberg and Lindenberger, 2020; Johansson et al., 2022).

Here, we established the same latent factor of GM structural integrity in a longitudinal design encompassing three time points. The assumption of factorial invariance across two groups and three time points was found to be tenable for the right and left hippocampus, right and left ACC, right PCC, left JLC, and right IFG. In the right and left ACC, right PCC, and left JLC, the ACG showed decreases in integrity, while the changes in the EG were significantly more positive, indicating that the exercise intervention had helped to maintain structural integrity in these areas. This supports the hypothesis that exercise has a neuroprotective effect on general structural integrity in older adults in areas of the brain where effects of exercise on volume have been observed before (e.g., Colcombe et al., 2006).

One mechanism hypothesized to underlie the relationship between aerobic exercise and brain structure is the increase in cerebral blood flow that occurs during bouts of exercise. In response to a complex combination of partial pressure of arterial carbon dioxide and oxygen, blood pressure, cerebral metabolism, and neurogenic regulation, acute physical exercise increases cerebral blood flow (Querido and Sheel, 2007; Smith and Ainslie, 2017), bringing with it oxygen and nutrients. With more resources available, both neurons and the surrounding cells may be better sustained, which would then be reflected in the latent factor of structural integrity. In contrast, individuals in the ACG, who did not engage in aerobic exercise, were less likely to experience this regular increase in cerebral blood flow, to the effect that GM structural integrity would be more likely to continue on a downward trajectory. Indeed, one study, a short-term exercise intervention (12 weeks) in older adults, even found an increase in cerebral blood flow at rest in the ACC within an exercise group vs. a control group (Chapman et al., 2013).

Notably, varying patterns of the timing of structural integrity changes were seen across ROIs. In the right ACC, the difference between groups in integrity change was seen in the first 3 months of the intervention, while in the left ACC and right PCC, this difference was seen in the second three months, and in the left JLC, this difference was evident throughout the six months. To some degree, group differences in change may have emerged only later in the study because unspecific initial interventions effects may have been shared across both conditions. The active control participants, though not engaging in exercise training, also changed their daily and weekly routines to incorporate more interaction with technology (tablet use), as well as with new social partners (weekly group sessions), which might have constituted a departure from their usual routine with potentially beneficial effects, in line with work suggesting positive associations between brain maintenance, cognition, and an active lifestyle (Lövdén et al., 2005; Hertzog et al., 2008; Nyberg et al., 2012; Small et al., 2012; Mintzer et al., 2019). However, to the degree that they habituated to these new daily practices, the initial overall effect might have worn off, while the mechanisms conveying a positive effect of exercise on brain integrity continued to operate in EG participants. Conversely, participants in the EG may have experienced initial maintenance in structural integrity at the beginning of their new training regimes, but as their brain and vascular systems grew more accustomed to the impulse afforded by aerobic exercise, a normal trajectory of decline might have resumed. Given the small sample size, these considerations are clearly speculative. More research is needed to better understand the cascade of mechanisms that convey benefits of aerobic exercise on different areas of the aging human brain.

### Positive Correlation Between Change in Cardiovascular Fitness and Structural Integrity in the Right Anterior Cingulate Cortex and Left Juxtapositional Lobule Cortex

Finally, in the right ACC and the left JLC, we were able to reliably measure significant individual differences in change, allowing us to investigate change-change correlations with cardiovascular fitness. A positive correlation was found between change in cardiovascular fitness and change in right ACC structural integrity from T1 to T2 in exercisers but not controls, indicating that those exercisers who gained more cardiovascular fitness during the intervention also showed less decline in structural integrity in the right ACC during the first 3 months of the intervention. A positive correlation was also found between change in cardiovascular fitness and change in left JLC structural integrity from T1 to T2, but this correlation did not differ between groups.

Many cross-sectional studies investigating the relationship between cardiovascular fitness and brain structure (using a single indicator approach) in older adults have found positive associations between fitness and in frontal areas, temporal areas, or both, as well as parietal, posterior (e.g., precuneus), and sub-cortical (e.g., caudate) areas (see review by d’Arbeloff, 2020). Similarly, some non-intervention longitudinal studies have reported positive associations between fitness and brain structure in similar brain regions (see d’Arbeloff, 2020), and one study found that baseline cardiovascular fitness was related to the progression of dementia severity and brain atrophy in Alzheimer’s patients (Vidoni et al., 2012). The current findings extend this previous work by reporting a change in the right ACC that is likely to reflect a causal effect of a change in cardiovascular fitness.

The current study has a number of limitations that should be addressed in future studies. First, the sample size was relatively small, especially for SEM. This might help to explain why individual differences in change often failed to differ reliably from zero. The current sample is also relatively homogeneous; healthy, previously sedentary older adults from an area with relatively high socioeconomic status were recruited to participate and were further screened for health conditions before being allowed to participate in the study. Healthy sedentary adults might be equipped with a range of protective factors that keep them healthy in the presence of a lifestyle that might result in deteriorating health in most other individuals. Thus, the generalizability of the present results to other segments of the aging population is unclear.

## Conclusion

In this study, we introduced a multimodal modeling approach for investigating the effects of aerobic fitness interventions on regional GM structural integrity in human aging. Our findings corroborate and extend earlier results by showing that at-home aerobic exercise among healthy sedentary older adults results in improved cardiovascular fitness and helps to maintain GM structural integrity in areas that have been found to show exercise-induced volume changes.

## Data Availability Statement

The original contributions presented in the study are publicly available. The data and relevant scripts for analysis can be found here: https://osf.io/yw865/.

## Ethics Statement

The studies involving human participants were reviewed and approved by the Ethics Committee of the German Psychological Society (DGPs). The participants provided their written informed consent to participate in this study.

## Author Contributions

SP assisted with data acquisition, analyzed the data, interpreted the results, and wrote the manuscript. MK preprocessed imaging data and revised the manuscript. YK and AB assisted with SEM analysis and revised the manuscript. NB designed the neuroimaging protocol and revised the manuscript. CM and JP performed physical assessments including cardiopulmonary exercise testing and revised the manuscript. BW designed the physical assessment protocol and revised the manuscript. SK designed the study and revised the manuscript. UL and SD designed the study, interpreted the results, and revised the manuscript. EW designed the study, preprocessed imaging data, interpreted the results, and revised the manuscript. All authors contributed to the article and approved the submitted version.

## Conflict of Interest

The authors declare that the research was conducted in the absence of any commercial or financial relationships that could be construed as a potential conflict of interest.

## Publisher’s Note

All claims expressed in this article are solely those of the authors and do not necessarily represent those of their affiliated organizations, or those of the publisher, the editors and the reviewers. Any product that may be evaluated in this article, or claim that may be made by its manufacturer, is not guaranteed or endorsed by the publisher.

## Footnotes

1
http://shared-reading.de/

2
www.fil.ion.ucl.ac.uk/spm

3
https://hmri-group.github.io/hMRI-toolbox/

4
https://neurovault.org/images/1705/

5
https://neurovault.org/images/1700/

## References

[B1] Ashburner J. Friston K. J. (2000). Voxel-Based Morphometry—The Methods. *NeuroImage* 11 805–821. 10.1006/nimg.2000.0582 10860804

[B2] Avants B. B. Tustison N. J. Song G. Cook P. A. Klein A. Gee J. C. (2011). A reproducible evaluation of ANTs similarity metric performance in brain image registration. *NeuroImage* 54 2033–2044. 10.1016/j.neuroimage.2010.09.025 20851191 PMC3065962

[B3] Avants B. B. Yushkevich P. Pluta J. Minkoff D. Korczykowski M. Detre J. (2010). The optimal template effect in hippocampus studies of diseased populations. *NeuroImage* 49 2457–2466. 10.1016/j.neuroimage.2009.09.062 19818860 PMC2818274

[B4] Beaulieu C. (2002). The basis of anisotropic water diffusion in the nervous system—A technical review. *NMR Biomed.* 15 435–455. 10.1002/nbm.782 12489094

[B5] Boker S. M. Neale M. C. Maes H. H. Wilde M. J. Spiegel M. Brick T. R. (2021). *OpenMx 2.19.6 User Guide*.*

[B6] Cabeza R. Albert M. Belleville S. Craik F. I. M. Duarte A. Grady C. L. (2018). Maintenance, reserve and compensation: The cognitive neuroscience of healthy ageing. *Nat. Rev. Neurosci.* 19 701–710. 10.1038/s41583-018-0068-2 30305711 PMC6472256

[B7] Chalmers R. P. Flora D. B. (2015). faoutlier: An R Package for Detecting Influential Cases in Exploratory and Confirmatory Factor Analysis. *Appl. Psychol. Measurement* 39 573–574. 10.1177/0146621615597894 29881028 PMC5978519

[B8] Chapman S. Aslan S. Spence J. DeFina L. Keebler M. Didehbani N. (2013). Shorter term aerobic exercise improves brain, cognition, and cardiovascular fitness in aging. *Front. Aging Neurosci.* 5:75. 10.3389/fnagi.2013.00075 24282403 PMC3825180

[B9] Colcombe S. J. Erickson K. I. Raz N. Webb A. G. Cohen N. J. McAuley E. (2003). Aerobic Fitness Reduces Brain Tissue Loss in Aging Humans. *J. Gerontol. Series A: Biol. Sci. Med. Sci.* 58 M176–M180. 10.1093/gerona/58.2.M176 12586857

[B10] Colcombe S. J. Erickson K. I. Scalf P. E. Kim J. S. Prakash R. McAuley E. (2006). Aerobic Exercise Training Increases Brain Volume in Aging Humans. *J. Gerontol. Series ABiol. Sci. Med. Sci.* 61 1166–1170. 10.1093/gerona/61.11.1166 17167157

[B11] Colcombe S. J. Kramer A. F. McAuley E. Erickson K. I. Scalf P. (2004). Neurocognitive Aging and Cardiovascular Fitness: Recent Findings and Future Directions. *J. Mol. Neurosci.* 24 009–014. 10.1385/JMN:24:1:009 15314244

[B12] R Core Team (2021). *R: A Language and Environment for Statistical Computing.* Vienna: R Foundation for Statistical Computing.

[B13] d’Arbeloff T. (2020). Cardiovascular fitness and structural brain integrity: An update on current evidence. *GeroScience* 42 1285–1306. 10.1007/s11357-020-00244-7 32767221 PMC7525918

[B14] Desikan R. S. Ségonne F. Fischl B. Quinn B. T. Dickerson B. C. Blacker D. (2006). An automated labeling system for subdividing the human cerebral cortex on MRI scans into gyral based regions of interest. *NeuroImage* 31 968–980. 10.1016/j.neuroimage.2006.01.021 16530430

[B15] Erickson K. I. Voss M. W. Prakash R. S. Basak C. Szabo A. Chaddock L. (2011). Exercise training increases size of hippocampus and improves memory. *Proc. Natl. Acad. Sci.* 108 3017–3022. 10.1073/pnas.1015950108 21282661 PMC3041121

[B16] Faul F. Erdfelder E. Buchner A. Lang A.-G. (2009). Statistical power analyses using G*Power 3.1: Tests for correlation and regression analyses. *Beh. Res. Methods* 41 1149–1160. 10.3758/BRM.41.4.1149 19897823

[B17] Faul F. Erdfelder E. Lang A.-G. Buchner A. (2007). G*Power 3: A flexible statistical power analysis program for the social, behavioral, and biomedical sciences. *Behav. Res. Methods* 39 175–191. 10.3758/bf03193146 17695343

[B18] Fjell A. M. Walhovd K. B. (2010). Structural Brain Changes in Aging: Courses. *Causes Cogn. Conseq. Rev. Neurosci.* 21 187–221. 10.1515/REVNEURO.2010.21.3.187 20879692

[B19] Fjell A. M. Walhovd K. B. Fennema-Notestine C. McEvoy L. K. Hagler D. J. Holland D. (2009). One-year brain atrophy evident in healthy aging. *J. Neurosci. J. Soc. Neurosci.* 29 15223–15231. 10.1523/JNEUROSCI.3252-09.2009 19955375 PMC2827793

[B20] Fukutomi H. Glasser M. F. Murata K. Akasaka T. Fujimoto K. Yamamoto T. (2019). Diffusion Tensor Model links to Neurite Orientation Dispersion and Density Imaging at high b-value in Cerebral Cortical Gray Matter. *Sci. Rep.* 9:12246. 10.1038/s41598-019-48671-7 31439874 PMC6706419

[B21] Gaser C. Dahnke R. (2016). CAT – A computational anatomy toolbox for the analysis of structural MRI data. *Hum. Brain Mapp.* 2016 336–348. 10.1093/gigascience/giae049 PMC11299546 39102518

[B22] Ghisletta P. Lindenberger U. (2004). Static and Dynamic Longitudinal Structural Analyses of Cognitive Changes in Old Age. *Gerontology* 50 12–16. 10.1159/000074383 14654721

[B23] Good C. D. Johnsrude I. S. Ashburner J. Henson R. N. A. Friston K. J. Frackowiak R. S. J. (2001). A Voxel-Based Morphometric Study of Ageing in 465 Normal Adult Human Brains. *NeuroImage* 14 21–36. 10.1006/nimg.2001.0786 11525331

[B24] Grydeland H. Walhovd K. B. Tamnes C. K. Westlye L. T. Fjell A. M. (2013). Intracortical Myelin Links with Performance Variability across the Human Lifespan: Results from T1- and T2-Weighted MRI Myelin Mapping and Diffusion Tensor Imaging. *J. Neurosci.* 33 18618–18630. 10.1523/JNEUROSCI.2811-13.2013 24259583 PMC6618798

[B25] Helms G. Dathe H. Dechent P. (2008a). Quantitative FLASH MRI at 3T using a rational approximation of the Ernst equation: Rational Approximation of the FLASH Signal. *Magnet. Resonan. Med.* 59 667–672. 10.1002/mrm.21542 18306368

[B26] Helms G. Dathe H. Kallenberg K. Dechent P. (2008b). High-resolution maps of magnetization transfer with inherent correction for RF inhomogeneity and T 1 relaxation obtained from 3D FLASH MRI: Saturation and Relaxation in MT FLASH. *Magnet. Resonan. Med.* 60 1396–1407. 10.1002/mrm.21732 19025906

[B27] HelpAge International. (2018). *Global AgeWatch Insights. The right to Health for Older People, the Right to be Counted*. Available online at: http://www.globalagewatch.org/download/5c0e922bebfcd

[B28] Hertzog C. Kramer A. F. Wilson R. S. Lindenberger U. (2008). Enrichment Effects on Adult Cognitive Development: Can the Functional Capacity of Older Adults Be Preserved and Enhanced? *Psychological science in the public interest*. *J. Am. Psychol. Soc.* 9 1–65. 10.1111/j.1539-6053.2009.01034.x 26162004

[B29] Hof P. R. Morrison J. H. (2004). The aging brain: Morphomolecular senescence of cortical circuits. *Trends Neurosci.* 27 607–613. 10.1016/j.tins.2004.07.013 15374672

[B30] Hunter M. D. (2018). State Space Modeling in an Open Source, Modular, Structural Equation Modeling Environment. Structural Equation Modeling. *Multidiscipl. J.* 25 307–324. 10.1080/10705511.2017.1369354

[B31] Jenkinson M. Beckmann C. F. Behrens T. E. J. Woolrich M. W. Smith S. M. (2012). FSL. *NeuroImage* 62 782–790. 10.1016/j.neuroimage.2011.09.015 21979382

[B32] Johansson J. Wåhlin A. Lundquist A. Brandmaier A. M. Lindenberger U. Nyberg L. (2022). Model of brain maintenance reveals specific change-change association between medial-temporal lobe integrity and episodic memory. *Aging Brain* 2:100027. 10.1016/j.nbas.2021.100027 PMC9999442 36908884

[B33] Kievit R. A. Brandmaier A. M. Ziegler G. van Harmelen A.-L. de Mooij S. M. M. Moutoussis M. (2018). Developmental cognitive neuroscience using latent change score models: A tutorial and applications. *Dev. Cogn. Neurosci.* 33 99–117. 10.1016/j.dcn.2017.11.007 29325701 PMC6614039

[B34] King A. C. (1991). Group- vs Home-Based Exercise Training in Healthy Older Men and Women: A Community-Based Clinical Trial. *JAMA* 266:1535. 10.1001/jama.1991.03470110081037 1880885

[B35] Kleemeyer M. M. Kühn S. Prindle J. Bodammer N. C. Brechtel L. Garthe A. (2016). Changes in fitness are associated with changes in hippocampal microstructure and hippocampal volume among older adults. *NeuroImage* 131 155–161. 10.1016/j.neuroimage.2015.11.026 26584869

[B36] Kline R. B. (2016). *Principles and Practice of Structural Equation Modeling (Fourth edition).* New York: The Guilford Press.

[B37] Köhncke Y. Düzel S. Sander M. C. Lindenberger U. Kühn S. Brandmaier A. M. (2021). Hippocampal and Parahippocampal Gray Matter Structural Integrity Assessed by Multimodal Imaging Is Associated with Episodic Memory in Old Age. *Cereb. Cortex* 31 1464–1477. 10.1093/cercor/bhaa287 33150357 PMC7869080

[B38] Kühn S. Düzel S. Eibich P. Krekel C. Wüstemann H. Kolbe J. (2017). In search of features that constitute an “enriched environment” in humans: Associations between geographical properties and brain structure. *Sci. Rep.* 7:11920. 10.1038/s41598-017-12046-7 28931835 PMC5607225

[B39] Lehmann M. Crutch S. J. Ridgway G. R. Ridha B. H. Barnes J. Warrington E. K. (2011). Cortical thickness and voxel-based morphometry in posterior cortical atrophy and typical Alzheimer’s disease. *Neurobiol. Aging* 32 1466–1476. 10.1016/j.neurobiolaging.2009.08.017 19781814

[B40] Lindenberger U. (2014). Human cognitive aging: Corriger la fortune? *Science* 346 572–578. 10.1126/science.1254403 25359964

[B41] Lövdén M. Ghisletta P. Lindenberger U. (2005). Social participation attenuates decline in perceptual speed in old and very old age. *Psychol. Aging* 20 423–434. 10.1037/0882-7974.20.3.423 16248702

[B42] Maass A. Düzel S. Goerke M. Becke A. Sobieray U. Neumann K. (2015). Vascular hippocampal plasticity after aerobic exercise in older adults. *Mol. Psychiatr.* 20 585–593. 10.1038/mp.2014.114 25311366

[B43] Mintzer J. Donovan K. A. Kindy A. Z. Lock S. L. Chura L. R. Barracca N. (2019). Lifestyle choices and brain health. *Front. Med.* 6:204. 10.3389/fmed.2019.00204 31637242 PMC6787147

[B44] Morey M. C. Dubbert P. M. Doyle M. E. MacAller H. Crowley G. M. Kuchibhatla M. (2003). From supervised to unsupervised exercise: factors associated with exercise adherence. *J. Aging. Phys. Act.* 11, 351–368. 10.1123/japa.11.3.351

[B45] Morrison J. H. Baxter M. G. (2012). The ageing cortical synapse: Hallmarks and implications for cognitive decline. *Nat. Rev. Neurosci.* 13 240–250. 10.1038/nrn3200 22395804 PMC3592200

[B46] Neale M. C. Hunter M. D. Pritikin J. N. Zahery M. Brick T. R. Kirkpatrick R. M. (2016). OpenMx 2.0: Extended Structural Equation and Statistical Modeling. *Psychometrika* 81 535–549. 10.1007/s11336-014-9435-8 25622929 PMC4516707

[B47] Nyberg L. Lindenberger U. (2020). ““Brain maintenance and cognition in old age,”,” in *The Cognitive Neurosciences*, 6th Edn, eds Poeppel D. Mangun G. R. Gazzaniga M. S. (Cambridge: MIT Press), 81–89.

[B48] Nyberg L. Lövdén M. Riklund K. Lindenberger U. Bäckman L. (2012). Memory aging and brain maintenance. *Trends Cogn. Sci.* 16 292–305. 10.1016/j.tics.2012.04.005 22542563

[B49] Nyberg L. Pudas S. (2019). Successful Memory Aging. *Ann. Rev. Psychol.* 70 219–243. 10.1146/annurev-psych-010418-103052 29949727

[B50] Pierpaoli C. Basser P. J. (1996). Toward a quantitative assessment of diffusion anisotropy. *Magnet. Resonan. Med.* 36 893–906. 10.1002/mrm.1910360612 8946355

[B51] Pritikin J. N. Hunter M. D. Boker S. M. (2015). Modular Open-Source Software for Item Factor Analysis. *Educ. Psychol. Measurement* 75 458–474. 10.1177/0013164414554615 27065479 PMC4822086

[B52] Querido J. S. Sheel A. W. (2007). Regulation of Cerebral Blood Flow During Exercise. *Sports Med.* 37 765–782. 10.2165/00007256-200737090-00002 17722948

[B53] Raz N. Daugherty A. M. (2018). Pathways to Brain Aging and Their Modifiers: Free-Radical-Induced Energetic and Neural Decline in Senescence (FRIENDS) Model - A Mini-Review. *Gerontology* 64 49–57. 10.1159/000479508 28858861 PMC5828941

[B54] Raz N. Lindenberger U. Rodrigue K. M. Kennedy K. M. Head D. Williamson A. (2005). Regional Brain Changes in Aging Healthy Adults: General Trends. *Individ. Diff. Modif. Cereb. Cortex* 15 1676–1689. 10.1093/cercor/bhi044 15703252

[B55] RStudio Team. (2021). *RStudio: Integrated Development Environment for R.* Boston: RStudio, PBC.

[B56] Ruscheweyh R. Willemer C. Krüger K. Duning T. Warnecke T. Sommer J. (2011). Physical activity and memory functions: An interventional study. *Neurobiol. Aging* 32 1304–1319. 10.1016/j.neurobiolaging.2009.08.001 19716631

[B57] Salvetti X. M. Oliveira J. A. Servantes D. M. Vincenzo de Paola A. A. (2008). How much do the benefits cost? Effects of a home-based training programme on cardiovascular fitness, quality of life, programme cost and adherence for patients with coronary disease. *Clin. Rehab.* 22 987–996. 10.1177/0269215508093331 18955430

[B58] Schermelleh-Engel K. Moosbrugger H. Müller H. (2003). Evaluating the Fit of Structural Equation Models: Tests of Significance and Descriptive Goodness-of-Fit Measures. *Methods Psychol. Res.* 8 23–74.

[B59] Seiler S. Ropele S. Schmidt R. (2014). Magnetization Transfer Imaging for *in vivo* Detection of Microstructural Tissue Changes in Aging and Dementia: A Short Literature Review. *J. Alzheimer’s Dis.* 42 S229–S237. 10.3233/JAD-132750 24840568

[B60] Small B. J. Dixon R. A. McArdle J. J. Grimm K. J. (2012). Do changes in lifestyle engagement moderate cognitive decline in normal aging? *Evid. Victoria Longitud. Stud. Neuropsychology* 26 144–155. 10.1037/a0026579 22149165 PMC3761970

[B61] Smith K. J. Ainslie P. N. (2017). Regulation of cerebral blood flow and metabolism during exercise: Cerebral blood flow and metabolism during exercise. *Exp. Physiol.* 102 1356–1371. 10.1113/EP086249 28786150

[B62] Smith S. M. Jenkinson M. Woolrich M. W. Beckmann C. F. Behrens T. E. J. Johansen-Berg H. (2004). Advances in functional and structural MR image analysis and implementation as FSL. *NeuroImage* 23 S208–S219. 10.1016/j.neuroimage.2004.07.051 15501092

[B63] Song S.-K. Yoshino J. Le T. Q. Lin S.-J. Sun S.-W. Cross A. H. (2005). Demyelination increases radial diffusivity in corpus callosum of mouse brain. *NeuroImage* 26 132–140. 10.1016/j.neuroimage.2005.01.028 15862213

[B64] Tabelow K. Balteau E. Ashburner J. Callaghan M. F. Draganski B. Helms G. (2019). HMRI – A toolbox for quantitative MRI in neuroscience and clinical research. *NeuroImage* 194 191–210. 10.1016/j.neuroimage.2019.01.029 30677501 PMC6547054

[B65] Tahedl M. (2018). *B.A.T.M.A.N.: Basic and Advanced Tractography with MRtrix for All Neurophiles.* OSF Home, 10.17605/OSF.IO/FKYHT

[B66] Tournier J.-D. Smith R. Raffelt D. Tabbara R. Dhollander T. Pietsch M. (2019). MRtrix3: A fast, flexible and open software framework for medical image processing and visualisation. *NeuroImage* 202:116137. 10.1016/j.neuroimage.2019.116137 31473352

[B67] Vidoni E. D. Honea R. A. Billinger S. A. Swerdlow R. H. Burns J. M. (2012). Cardiorespiratory fitness is associated with atrophy in Alzheimer’s and aging over 2 years. *Neurobiol. Aging* 33 1624–1632. 10.1016/j.neurobiolaging.2011.03.016 21531480 PMC3156963

[B68] Walhovd K. B. Westerhausen R. de Lange A.-M. G. Bråthen A. C. S. Grydeland H. Engvig A. (2016). Premises of plasticity — And the loneliness of the medial temporal lobe. *NeuroImage* 131 48–54. 10.1016/j.neuroimage.2015.10.060 26505299

[B69] Wansbeek T. Meijer E. (2001). ““Measurement error and latent variables,”,” in *A Companion to Theoretical Econometrics*, ed. Baltagi B. H. (New Jersey: Wiley), 162–179.

[B70] Weinstein A. M. Voss M. W. Prakash R. S. Chaddock L. Szabo A. White S. M. (2012). The association between aerobic fitness and executive function is mediated by prefrontal cortex volume. *BrainBehav., Immun.* 26 811–819. 10.1016/j.bbi.2011.11.008 22172477 PMC3321393

[B71] Weiskopf N. Lutti A. Helms G. Novak M. Ashburner J. Hutton C. (2011). Unified segmentation based correction of R1 brain maps for RF transmit field inhomogeneities (UNICORT). *NeuroImage* 54 2116–2124. 10.1016/j.neuroimage.2010.10.023 20965260 PMC3018573

[B72] Weiskopf N. Suckling J. Williams G. Correia M. M. Inkster B. Tait R. (2013). Quantitative multi-parameter mapping of R1. PD*, MT, and R2* at 3T: *A multi-center validation*. *Front. Neurosci.* 7: 95. 10.3389/fnins.2013.00095 23772204 PMC3677134

[B73] Wenger E. Düzel S. Kleemeyer M. M. Polk S. E. Köhncke Y. Bodammer N. C. (2022). Vamos en bici: Study protocol of an investigation of cognitive and neural changes following language training, physical exercise training, or a combination of both. *BioRxiv* [Preprint]. 10.1101/2022.01.30.478181

[B74] Woolrich M. W. Jbabdi S. Patenaude B. Chappell M. Makni S. Behrens T. (2009). Bayesian analysis of neuroimaging data in FSL. *NeuroImage* 45 S173–S186. 10.1016/j.neuroimage.2008.10.055 19059349

[B75] Zatorre R. J. Fields R. D. Johansen-Berg H. (2012). Plasticity in gray and white: Neuroimaging changes in brain structure during learning. *Nat. Neurosci.* 15 528–536. 10.1038/nn.3045 22426254 PMC3660656

