# Supplementary material for: Change in Latent Gray-Matter Structural Integrity Is Associated With Change in Cardiovascular Fitness in Older Adults Who Engage in At-Home Aerobic Exercise
Source: Front Hum Neurosci. 2022 May 17;16:852737. doi: 10.3389/fnhum.2022.852737 (PMC9152142; doi:10.3389/fnhum.2022.852737)
Supplement: Supplementary file 1 [file Data_Sheet_1.docx]

## S1: Supplementary methods

**Statistical analyses**

### Magnetic resonance imaging

**Acquisition.** T_1_-weighted images were obtained using a 3D T_1_-weighted magnetization prepared gradient-echo (MPRAGE) sequence using the following parameters: repetition time (TR) = 2500 ms; echo time (TE) = 4.77 ms; inversion time (TI) = 1100 ms; flip angle = 7°; acquisition matrix = 256 × 256 × 192; 1 mm^3^ isotropic voxels; with the prescan normalize option and a 3D distortion correction for non-linear gradients; acquisition time = 9:20 min.

The multi-parameter mapping protocol used to acquire the MT maps comprised one static magnetic (B_0_) gradient echo (GRE)-field map, one radiofrequency (RF) transmit field map (B_1_), and three multi-echo 3D fast low angle shot (FLASH) scans (Helms, Dathe, & Dechent, 2008; Tabelow et al., 2019). The B_0_ GRE-field mapping sequence was acquired with the following parameters: 64 transversal slices; slice thickness = 2 mm with 50% distance factor; TR = 1020 ms; TE1/TE2 = 10/12.46 ms; flip angle = 90°; acquisition matrix = 64 × 64; FOV = 192 × 192 mm; right-left phase encoding direction; bandwidth (BW) = 260 Hz/pixel; flow compensation; 3.0 × 3.0 × 2.0 mm^3^ voxel size; acquisition time = 2:14 min.

Maps of the local RF transmit/B_1_^+^ field were acquired following recommendations by Lutti and colleagues (2010) and were measured and estimated from a 3D EPI acquisition of spin and stimulated echoes (SE and STE) with different flip angles. The following parameters were used: 4 mm isotropic resolution, matrix = 64 × 48 × 48, FOV = 256 × 192 × 192 mm, parallel imaging using GRAPPA factor 2 × 2 in PE and partition directions, TR = 500 ms, TE_SE/STE_/mixing time = 39.06 ms/33.80 ms. Eleven pairs of SE/STE image volumes were measured successively employing decreasing flip angles α from 115° to 65° in steps of –5° (applied in a α–2α–α series of RF pulses to produce SEs and STEs; see Akoka et al., 1993). Acquisition time was 3 min.

The three different multi-echo FLASH sequences were acquired with predominant T_1_ weighting (T_1_w), proton density weighting (PDw), or magnetization transfer weighting (MTw) by adjusting the repetition time (TR) and flip angle (α; T_1_w: TR/α = 24.5 ms/21°; PDw and MTw: TR/α = 24.5 ms/6°) and applying an off-resonance Gaussian-shaped RF pulse (4 ms duration, 220° nominal flip angle, 2 kHz frequency offset from water resonance); for the MTw sequence this was applied prior to excitation. Multiple gradient echoes with alternating readout polarity were acquired at six equidistant echo times (TE) between 2.34 ms and 14.04 ms for the T_1_w and MTw acquisitions, with two additional echoes at TE = 16.38 ms and 18.72 ms for the PDw acquisition. A high readout bandwidth (BW) of 465 Hz/pixel was used to minimize off-resonance artifacts. To reduce data acquisition time, GRAPPA parallel imaging with an acceleration factor of two was applied in the phase-encoding direction (anterior-posterior; outer/slow phase encoding loop) and 6/8 partial Fourier acquisitions in the partition direction (left-right; inner/fast phase encoding loop). The following additional acquisition parameters were used: 1 mm isotropic resolution, 176 slices per slab, FOV = 256 × 240 mm, acquisition time of each FLASH sequence = 7:03 min.

Diffusion-weighted images were obtained with a single-shot diffusion-weighted spin-echo-refocused echo-planar imaging sequence with the following parameters: TR = 9700 ms; TE = 120 ms; 62 slices; FOV = 224 × 224 mm; a two-shell scheme was used for diffusion-weighting applying two *b*-values: 710 s/mm^2^ (30 directions) and 2850 s/mm^2^ (60 directions), with directions distributed over a whole sphere for each shell, plus ten non-diffusion-weighted images; GRAPPA acceleration factor = 2; 2 mm^3^ isotropic voxels; acquisition time = 16:41 min. Six additional images inverting the phase encoding direction were acquired without diffusion weighting; acquisition time = 1:29 min.

**Preprocessing.** Structural T_1_-weighted images were preprocessed using the Computational Anatomy Toolbox 12 (CAT12, Structural Brain Mapping group, Jena University Hospital; Gaser & Dahnke, 2016) in Statistical Parametric Mapping (SPM12, Institute of Neurology; www.fil.ion.ucl.ac.uk/spm) using the default parameters of the longitudinal pipeline, in which an individual participant’s images first undergo an inverse-consistent realignment (including intra-subject bias correction) and a mean image is calculated. Spatial normalization parameters are then estimated using Dartel normalization based on the segmentations of the mean image. These normalization parameters are then applied to the segmentations of the images at all time points. Nonlinear-only modulation of gray and white matter segments was applied. These images were smoothed using an 8 mm full-width half-maximum (FWHM) standard Gaussian kernel.

Estimation of MT maps was conducted in SPM12 using the hMRI toolbox (Tabelow et al., 2019; https://hmri-group.github.io/hMRI-toolbox/). Within this toolbox, quantitative as well as semi-quantitative estimates of MT, PD, R1, and R2* were computed from unprocessed multi-echo T_1_w, PDw, and MTw RF-spoiled gradient echo acquisitions using the *Create hMRI maps* module, which corrects the qMRI estimates for spatial receive and transmit field inhomogeneities. As has been described in more detail elsewhere (Helms, Dathe, & Dechent, 2008; Helms, Dathe, Kallenberg, et al., 2008; Weiskopf et al., 2011, 2013), the signal from the multi-echo T_1_w, PDw, and MTw echoes can be described by the Ernst equation (Ernst & Anderson, 1966; Helms, Dathe, & Dechent, 2008; Helms, Dathe, Kallenberg, et al., 2008).

The effective transverse relaxation rate (R2* = 1/T_2_*) was derived from the TE dependence of the signal. The unified description of the multi-echo data from all three contrasts into a single signal model, or ESTATICS (Weiskopf et al., 2014), provides a more robust estimation of R2* with a higher signal-to-noise ratio. Using approximations of the signal equations for small repetition time TR and small flip angle, the longitudinal relaxation rate (R1), A* map (proportional to PD), and MT were estimated. PD maps were then computed by calibrating the mean PD value in white matter to 69 percent units, since the global mean PD cannot be estimated accurately.

A corrected MT saturation value was calculated by correcting the original MT value by the local RF transmit field. This semi-quantitative parameter is unaffected by R1 and RF transmit field variations, in contrast to the conventional MT ratio (Helms, Dathe, Kallenberg, et al., 2008). As implemented in the toolbox, *Unified Segmentation* was used to correct for RF sensitivity bias and applied the recommended 3D EPI B1 bias correction (Lutti et al., 2012).

A longitudinal processing pipeline of the data was adapted to achieve an improved within-subject coregistration of the created maps. In this pipeline, all MT and PD maps were first thresholded (MT: 0–5, PD: 0–200) to improve segmentation performance. Multichannel segmentations were then conducted using the thresholded MT and PD maps at all measurement time points (up to three per participant). The resulting gray and white matter segmentations from all three measurements per subject, formatted to be imported to DARTEL using the *DARTEL imported* option, were then fed into *SHOOT* to create an unbiased within-subject registration. The respective deformations were applied to the raw MT and PD maps to warp them into each subject’s template space, and the median MT and PD maps across all measurements of a single individual were computed. These median maps were then subjected to a multichannel segmentation again and a group template was created from the resulting DARTEL-imported gray and white matter segmentations of all subjects using *SHOOT*. Subsequently, the two deformation fields (from native space to subject template space and from subject template space to group template space) were combined. For each measurement, this combined deformation field together with all four parameter maps (MT, PD, R1, R2*) was fed into *SHOOT normalize* to achieve normalization to MNI space. The gray and white matter segmentations derived by the multichannel segmentation of the median MT and PD maps were also spatially normalized to MNI space using Jacobian modulation. Finally, from these normalized tissue class segmentations as well as the four parameter maps, smoothed tissue-specific MPMs were computed applying a 6mm FWHM smoothing kernel and weighted averaging.

DW images were preprocessed using MRtrix (version 3.0_RC3; Tournier et al., 2019), FSL (FMRIB's Software Library, version 6.0.2; Jenkinson et al., 2012; Smith et al., 2004; Woolrich et al., 2009), and ANTS (version 2.2.0; Avants et al., 2010, 2011), following the Basic and Advanced Tractography with MRtrix for All Neurophiles (B.A.T.M.A.N.) tutorial (Tahedl, 2018). DW images were first denoised using *dwidenoise* with a 5 × 5 × 5 patch size, Gibb’s ringing artifacts were removed using *mrdegibbs*, correction for EPI distortion, B_0_-field inhomogeneity, and eddy-current and movement distortion were applied using FSL’s *topup* and *eddy cuda* with the default settings, adding outlier detection and replacement, and saving contrast-to-noise ratio and residual maps. Binary brain masks were created by running FSL’s *bet* on each participant’s mean b0 image. These were visually checked and manually fixed in cases where needed. Diffusion tensor maps were fit to each dataset using the lower *b*-value (710 s/mm^2^) with the MRtrix command *dwi2tensor* and afterwards MD values were derived via *tensor2metric*.

### Modeling changes in fitness

**Group mean differences in change in VO_2_peak.** Latent change score models (LCSM) were used to evaluate group mean differences in change in VO_2_peak and GM integrity following the tutorial by Kievit and colleagues (2018). A univariate LCSM was built to measure mean change in VO_2_peak, in which a pseudo-latent factor, ΔVO_2_peak, captured the difference in VO_2_peak between T1 and T3, which was in this case simply a difference score, as VO_2_peak was directly measured. The means and variances of VO_2_peak at T1 and ΔVO_2_peak were estimated, as well as the correlation between the two. To test whether the EG showed significantly more positive mean change than the ACG, a multigroup model was used to test whether the change means could be fixed to equality across groups without significantly affecting the model fit.

### Modeling changes in GM structural integrity

**Factorial invariance testing of GM integrity models.** GM integrity was modeled as a latent variable with freely estimated mean and variance in each of the pre-selected ROIs separately. This latent variable predicted VBM (loading fixed to 1 for model identification), MT (loading freely estimated), and MD (loading freely estimated), each with freely estimated residual variance. To ensure that the latent factor measured represented the same construct across groups as well as across time points, a series of measurement invariance tests were conducted sequentially, first across groups at T1 then across time points, collapsing the two groups. First, the GM integrity factor structure at T1 was tested for metric (i.e., identical factor loadings) then strict factorial invariance (i.e., identical residual variances) across the two groups using LRTs (Cheung & Rensvold, 1999). In ROIs showing strict group invariance, the two groups were collapsed, and factorial invariance across the three time points was tested by including the same measurement structure of GM integrity at T2 and T3 in an unstructured model with freely estimated covariance between latent integrity at each time point (Widaman et al., 2010), as well as residual covariances of each image modality (T1 to T2, T2 to T3, and T1 to T3), set to be equal within each modality. Residual means were set to zero under the assumption that changes in the latent integrity factor would reflect changes in the three observed variables similarly; that is, the pattern of change over time was assumed to be similar across observed variables. Further, none of the models showed a worse fit when assuming residual means of zero versus freely estimated, thus we omit them for the sake of parsimony.

**Group differences in mean change in GM integrity.** For those regions showing strict factorial invariance across time (i.e., identical factor loadings and residual variances), to investigate group differences in mean change, the unstructured latent matrix was then replaced with a structured matrix modeling latent change scores from T1 to T2 and from T2 to T3. This allowed the models to have different means and variances in change between each pair of consecutive time points. Covariances between T1 and both change from T1 to T2 and from T2 to T3 and the covariance between the two latent change scores were freely estimated. Differences in group mean change were then tested using a multigroup model; an LRT was conducted between a model in which the means were freely estimated within each group and a model in which the means were restricted to be equal across groups.

**Change-change covariance between VO_2_peak and GM integrity.** Lastly, the relationship between change in VO_2_peak and change in latent GM integrity was investigated using a bivariate LCSM, in which the covariance between latent change score variables was estimated (VO_2_peak change to GM integrity change from T1 to T2, VO_2_peak change to GM integrity change from T2 to T3), accounting for the covariance between the two baseline variables (VO_2_peak and latent GM integrity at T1). See Figure 2 for the full bivariate LCSM. First, to confirm that reliable individual differences in change in GM integrity could be detected in the full multivariate model, which is necessary to investigate change-change relationships, two separate LRTs were conducted between the full model and 1) a model in which the variance of change in integrity from T1 to T2 and all covariance parameters connected with change in integrity from T1 to T2 were fixed to zero (*df* = 4), and 2) a model in which the variance of change in integrity from T2 to T3 and all covariance parameters connected with change in integrity from T2 to T3 were fixed to zero (*df* = 4) following the logic of the generalized variance test (e.g., Brandmaier et al., 2018). To investigate whether the covariance between change in cardiovascular fitness and change in GM integrity was significant, the full model including both groups was compared to a constrained nested model in which one covariance path was fixed to zero. Finally, an LRT was run to investigate whether the covariance paths could be set to equal across groups without significantly affecting model fit, and if so, LRTs were used to test whether the covariances were greater than zero in each group separately.

References

Akoka, S., Franconi, F., Seguin, F., & Le Pape, A. (1993). Radiofrequency map of an NMR coil by imaging. *Magnetic Resonance Imaging*, *11*(3), 437–441. doi: 10.1016/0730-725X(93)90078-R

Brandmaier, A. M., von Oertzen, T., Ghisletta, P., Lindenberger, U., & Hertzog, C. (2018). Precision, Reliability, and Effect Size of Slope Variance in Latent Growth Curve Models: Implications for Statistical Power Analysis. *Frontiers in Psychology*, *9*, 294. doi: 10.3389/fpsyg.2018.00294

Cheung, G. W., & Rensvold, R. B. (1999). Testing Factorial Invariance across Groups: A Reconceptualization and Proposed New Method. *Journal of Management*, *25*(1), 1–27. doi: 10.1177/014920639902500101

Ernst, R. R., & Anderson, W. A. (1966). Application of Fourier Transform Spectroscopy to Magnetic Resonance. *Review of Scientiﬁc Instruments*, *37*, 93–102.

Gaser, C., & Dahnke, R. (2016). CAT – A computational anatomy toolbox for the analysis of structural MRI data. *Human brain mapping, 2016*, 336-348.

Helms, G., Dathe, H., & Dechent, P. (2008). Quantitative FLASH MRI at 3T using a rational approximation of the Ernst equation: Rational Approximation of the FLASH Signal. *Magnetic Resonance in Medicine*, *59*(3), 667–672. doi: 10.1002/mrm.21542

Helms, G., Dathe, H., Kallenberg, K., & Dechent, P. (2008). High-resolution maps of magnetization transfer with inherent correction for RF inhomogeneity and *T* _1_ relaxation obtained from 3D FLASH MRI: Saturation and Relaxation in MT FLASH. *Magnetic Resonance in Medicine*, *60*(6), 1396–1407. doi: 10.1002/mrm.21732

Jenkinson, M., Beckmann, C. F., Behrens, T. E. J., Woolrich, M. W., & Smith, S. M. (2012). FSL. *NeuroImage*, *62*(2), 782–790. doi: 10.1016/j.neuroimage.2011.09.015

Kievit, R. A., Brandmaier, A. M., Ziegler, G., van Harmelen, A.-L., de Mooij, S. M. M., Moutoussis, M., Goodyer, I. M., Bullmore, E., Jones, P. B., Fonagy, P., Lindenberger, U., & Dolan, R. J. (2018). Developmental cognitive neuroscience using latent change score models: A tutorial and applications. *Developmental Cognitive Neuroscience*, *33*, 99–117. doi: 10.1016/j.dcn.2017.11.007

Lutti, A., Hutton, C., Finsterbusch, J., Helms, G., & Weiskopf, N. (2010). Optimization and validation of methods for mapping of the radiofrequency transmit field at 3T: Optimized RF Transmit Field Mapping at 3T. *Magnetic Resonance in Medicine*, *64*(1), 229–238. doi: 10.1002/mrm.22421

Lutti, A., Stadler, J., Josephs, O., Windischberger, C., Speck, O., Bernarding, J., Hutton, C., & Weiskopf, N. (2012). Robust and Fast Whole Brain Mapping of the RF Transmit Field B1 at 7T. *PLoS ONE*, *7*(3), e32379. doi: 10.1371/journal.pone.0032379

Smith, S. M., Jenkinson, M., Woolrich, M. W., Beckmann, C. F., Behrens, T. E. J., Johansen-Berg, H., Bannister, P. R., De Luca, M., Drobnjak, I., Flitney, D. E., Niazy, R. K., Saunders, J., Vickers, J., Zhang, Y., De Stefano, N., Brady, J. M., & Matthews, P. M. (2004). Advances in functional and structural MR image analysis and implementation as FSL. *NeuroImage*, *23*, S208–S219. doi: 10.1016/j.neuroimage.2004.07.051

Tabelow, K., Balteau, E., Ashburner, J., Callaghan, M. F., Draganski, B., Helms, G., Kherif, F., Leutritz, T., Lutti, A., Phillips, C., Reimer, E., Ruthotto, L., Seif, M., Weiskopf, N., Ziegler, G., & Mohammadi, S. (2019). HMRI – A toolbox for quantitative MRI in neuroscience and clinical research. *NeuroImage*, *194*, 191–210. doi: 10.1016/j.neuroimage.2019.01.029

Tahedl, M. (2018). *B.A.T.M.A.N.: Basic and Advanced Tractography with MRtrix for All Neurophiles*. doi: 10.17605/OSF.IO/FKYHT

Tournier, J.-D., Smith, R., Raffelt, D., Tabbara, R., Dhollander, T., Pietsch, M., Christiaens, D., Jeurissen, B., Yeh, C.-H., & Connelly, A. (2019). MRtrix3: A fast, flexible and open software framework for medical image processing and visualisation. *NeuroImage*, *202*, 116137. doi: 10.1016/j.neuroimage.2019.116137

Weiskopf, N., Callaghan, M. F., Josephs, O., Lutti, A., & Mohammadi, S. (2014). Estimating the apparent transverse relaxation time (R2*) from images with different contrasts (ESTATICS) reduces motion artifacts. *Frontiers in Neuroscience*, *8*. doi: 10.3389/fnins.2014.00278

Weiskopf, N., Lutti, A., Helms, G., Novak, M., Ashburner, J., & Hutton, C. (2011). Unified segmentation based correction of R1 brain maps for RF transmit field inhomogeneities (UNICORT). *NeuroImage*, *54*(3), 2116–2124. doi: 10.1016/j.neuroimage.2010.10.023

Weiskopf, N., Suckling, J., Williams, G., Correia, M. M., Inkster, B., Tait, R., Ooi, C., Bullmore, E. T., & Lutti, A. (2013). Quantitative multi-parameter mapping of R1, PD*, MT, and R2* at 3T: A multi-center validation. *Frontiers in Neuroscience*, *7*. doi: 10.3389/fnins.2013.00095

Widaman, K. F., Ferrer, E., & Conger, R. D. (2010). Factorial Invariance Within Longitudinal Structural Equation Models: Measuring the Same Construct Across Time. *Child Development Perspectives*, *4*(1), 10–18. doi: 10.1111/j.1750-8606.2009.00110.x

Woolrich, M. W., Jbabdi, S., Patenaude, B., Chappell, M., Makni, S., Behrens, T., Beckmann, C., Jenkinson, M., & Smith, S. M. (2009). Bayesian analysis of neuroimaging data in FSL. *NeuroImage*, *45*(1), S173–S186. doi: 10.1016/j.neuroimage.2008.10.055

## S2: Supplementary results

**Bilateral regions of interest**

ROIs were also averaged across left and right hemispheres. Out of the six bilateral GM structural integrity models, two survived testing for factorial invariance: hippocampus and PCC. For these models, all standardized factor loadings were significant (*p*s < .050; hippocampus: *λ*_MD_ = –0.941, *λ*_MT_ = 0.532; PCC: *λ*_MD_ = –0.931, *λ*_MT_ = 0.471). No differences in mean group change were detected in the hippocampus. A significant maintenance effect of exercise in the bilateral PCC from T2 to T3 was found, Δ*χ^2^*(1) = 4.80, *p* = .014, with the EG showing no mean change in PCC integrity, *β* = –0.145, *SE* = 0.357, Δ*χ^2^*(1) = 0.17, *p* = .339, while the ACG showed a significant mean decrease, *β* = –1.161, *SE* = 0.597, Δ*χ^2^*(1) = 10.09, *p* = .001. Neither model showed adequate variance in change to test for change-change correlations with cardiovascular fitness.

## S3: Means of observed variables

|  | ACG | | |  | EG | | |
| --- | --- | --- | --- | --- | --- | --- | --- |
| Observed variable | T1 | T2 | T3 |  | T1 | T2 | T3 |
| VO_2_peak | 22.8 | — | 24.2 |  | 23.8 | — | 25.5 |
| VBM HC right | 0.433 | 0.432 | 0.434 |  | 0.429 | 0.432 | 0.427 |
| VBM HC left | 0.444 | 0.445 | 0.446 |  | 0.445 | 0.450 | 0.448 |
| VBM ACC right | 0.391 | 0.387 | 0.385 |  | 0.392 | 0.392 | 0.391 |
| VBM ACC left | 0.404 | 0.401 | 0.398 |  | 0.389 | 0.390 | 0.389 |
| VBM PCC right | 0.390 | 0.387 | 0.385 |  | 0.369 | 0.367 | 0.367 |
| VBM PCC left | 0.405 | 0.404 | 0.401 |  | 0.386 | 0.383 | 0.382 |
| VBM PCG right | 0.317 | 0.314 | 0.308 |  | 0.308 | 0.307 | 0.304 |
| VBM PCG left | 0.317 | 0.315 | 0.308 |  | 0.315 | 0.313 | 0.309 |
| VBM JLC right | 0.366 | 0.363 | 0.360 |  | 0.361 | 0.355 | 0.355 |
| VBM JLC left | 0.366 | 0.362 | 0.359 |  | 0.356 | 0.353 | 0.353 |
| VBM IFG right | 0.350 | 0.347 | 0.347 |  | 0.348 | 0.357 | 0.356 |
| VBM IFG left | 0.349 | 0.351 | 0.348 |  | 0.349 | 0.355 | 0.354 |
| MT HC right | 0.805 | 0.815 | 0.811 |  | 0.817 | 0.824 | 0.803 |
| MT HC left | 0.804 | 0.807 | 0.811 |  | 0.813 | 0.822 | 0.798 |
| MT ACC right | 0.822 | 0.827 | 0.812 |  | 0.824 | 0.831 | 0.816 |
| MT ACC left | 0.819 | 0.828 | 0.811 |  | 0.819 | 0.826 | 0.810 |
| MT PCC right | 0.862 | 0.864 | 0.851 |  | 0.864 | 0.873 | 0.867 |
| MT PCC left | 0.865 | 0.871 | 0.857 |  | 0.870 | 0.878 | 0.876 |
| MT PCG right | 0.879 | 0.880 | 0.868 |  | 0.877 | 0.882 | 0.876 |
| MT PCG left | 0.869 | 0.875 | 0.860 |  | 0.873 | 0.869 | 0.864 |
| MT JLC right | 0.824 | 0.836 | 0.823 |  | 0.822 | 0.821 | 0.812 |
| MT JLC left | 0.828 | 0.845 | 0.830 |  | 0.831 | 0.832 | 0.822 |
| MT IFG right | 0.828 | 0.834 | 0.825 |  | 0.826 | 0.830 | 0.829 |
| MT IFG left | 0.821 | 0.836 | 0.823 |  | 0.831 | 0.828 | 0.821 |
| MD HC right | 0.00170 | 0.00169 | 0.00171 |  | 0.00165 | 0.00159 | 0.00160 |
| MD HC left | 0.00175 | 0.00174 | 0.00174 |  | 0.00169 | 0.00162 | 0.00164 |
| MD ACC right | 0.00139 | 0.00140 | 0.00140 |  | 0.00132 | 0.00128 | 0.00130 |
| MD ACC left | 0.00157 | 0.00158 | 0.00159 |  | 0.00153 | 0.00152 | 0.00152 |
| MD PCC right | 0.00130 | 0.00132 | 0.00134 |  | 0.00130 | 0.00132 | 0.00132 |
| MD PCC left | 0.00144 | 0.00146 | 0.00146 |  | 0.00140 | 0.00142 | 0.00142 |
| MD PCG right | 0.00164 | 0.00167 | 0.00169 |  | 0.00164 | 0.00166 | 0.00166 |
| MD PCG left | 0.00163 | 0.00167 | 0.00168 |  | 0.00163 | 0.00164 | 0.00165 |
| MD JLC right | 0.00155 | 0.00159 | 0.00160 |  | 0.00158 | 0.00161 | 0.00161 |
| MD JLC left | 0.00173 | 0.00176 | 0.00178 |  | 0.00174 | 0.00177 | 0.00179 |
| MD IFG right | 0.00184 | 0.00188 | 0.00187 |  | 0.00182 | 0.00179 | 0.00181 |
| MD IFG left | 0.00180 | 0.00181 | 0.00182 |  | 0.00177 | 0.00176 | 0.00177 |
| *Note.* ACG = active control group, EG = exercise group, T1 = time point 1 (0 months), T2 = time point 2 (3 months), T3 = time point 3 (6 months), VO_2_peak = peak oxygen uptake, VBM = voxel-based morphometry, MT = magnetization transfer, MD = mean diffusivity, HC = hippocampus, ACC = anterior cingulate cortex, PCC = posterior cingulate cortex, PCG = precentral gyrus, JLC = juxtapositional lobule cortex, IFG = inferior frontal gyrus. | | | | | | | |

## S4: Pearson correlation coefficients between observed variables at T1

| Observed variable | 1. | 2. | 3. | 4. | 5. | 6. | 7. | 8. | 9. | 10. | 11. | 12. | 13. | 14. | 15. | 16. | 17. | 18. | 19. | 20. | 21. | 22. | 23. | 24. | 25. | 26. | 27. | 28. | 29. | 30. | 31. | 32. | 33. | 34. | 35. | 36. |
| --- | --- | --- | --- | --- | --- | --- | --- | --- | --- | --- | --- | --- | --- | --- | --- | --- | --- | --- | --- | --- | --- | --- | --- | --- | --- | --- | --- | --- | --- | --- | --- | --- | --- | --- | --- | --- |
| 1. VO2peak |  |  |  |  |  |  |  |  |  |  |  |  |  |  |  |  |  |  |  |  |  |  |  |  |  |  |  |  |  |  |  |  |  |  |  |  |
| 2. VBM HC right | –.11 |  |  |  |  |  |  |  |  |  |  |  |  |  |  |  |  |  |  |  |  |  |  |  |  |  |  |  |  |  |  |  |  |  |  |  |
| 3. MT HC right | .13 | **.35*** |  |  |  |  |  |  |  |  |  |  |  |  |  |  |  |  |  |  |  |  |  |  |  |  |  |  |  |  |  |  |  |  |  |  |
| 4. MD HC right | .01 | **–.71*** | **–.43*** |  |  |  |  |  |  |  |  |  |  |  |  |  |  |  |  |  |  |  |  |  |  |  |  |  |  |  |  |  |  |  |  |  |
| 5. VBM HC left | –.01 | .82* | .19 | –.64* |  |  |  |  |  |  |  |  |  |  |  |  |  |  |  |  |  |  |  |  |  |  |  |  |  |  |  |  |  |  |  |  |
| 6. MT HC left | .17 | .38* | .84* | –.44* | **.32*** |  |  |  |  |  |  |  |  |  |  |  |  |  |  |  |  |  |  |  |  |  |  |  |  |  |  |  |  |  |  |  |
| 7. MD HC left | –.05 | –.68* | –.32* | .89* | **–.75*** | **–.40*** |  |  |  |  |  |  |  |  |  |  |  |  |  |  |  |  |  |  |  |  |  |  |  |  |  |  |  |  |  |  |
| 8. VBM ACC right | –.12 | .32* | .05 | –.36* | .37* | –.09 | –.35* |  |  |  |  |  |  |  |  |  |  |  |  |  |  |  |  |  |  |  |  |  |  |  |  |  |  |  |  |  |
| 9. MT ACC right | .11 | .31* | .50* | –.30* | .20 | .41* | –.27* | **.18** |  |  |  |  |  |  |  |  |  |  |  |  |  |  |  |  |  |  |  |  |  |  |  |  |  |  |  |  |
| 10. MD ACC right | –.01 | –.43* | –.21 | .41* | –.36* | –.16 | .46* | **–.52*** | **–.51*** |  |  |  |  |  |  |  |  |  |  |  |  |  |  |  |  |  |  |  |  |  |  |  |  |  |  |  |
| 11. VBM ACC left | –.01 | .37* | –.03 | –.30* | .45* | –.10 | –.36* | .68* | .04 | –.27* |  |  |  |  |  |  |  |  |  |  |  |  |  |  |  |  |  |  |  |  |  |  |  |  |  |  |
| 12. MT ACC left | .12 | .29* | .44* | –.28* | .18 | .37* | –.24 | .11 | .94* | –.42* | **.06** |  |  |  |  |  |  |  |  |  |  |  |  |  |  |  |  |  |  |  |  |  |  |  |  |  |
| 13. MD ACC left | –.02 | –.22 | –.17 | .29* | –.21 | –.11 | .32* | –.21 | –.41* | .65* | **–.26*** | **–.44*** |  |  |  |  |  |  |  |  |  |  |  |  |  |  |  |  |  |  |  |  |  |  |  |  |
| 14. VBM PCC right | .08 | .36* | .08 | –.35* | .33* | –.08 | –.31* | .42* | .13 | –.27* | .55* | .11 | –.23* |  |  |  |  |  |  |  |  |  |  |  |  |  |  |  |  |  |  |  |  |  |  |  |
| 15. MT PCC right | .08 | .25* | .77* | –.30* | .09 | .59* | –.21 | .19 | .73* | –.34* | .03 | .67* | –.31* | **.12** |  |  |  |  |  |  |  |  |  |  |  |  |  |  |  |  |  |  |  |  |  |  |
| 16. MD PCC right | .16 | –.39* | –.18 | .28* | –.32* | –.06 | .28* | –.46* | –.45* | .71* | –.35* | –.40* | .67* | **–.45*** | **–.39*** |  |  |  |  |  |  |  |  |  |  |  |  |  |  |  |  |  |  |  |  |  |
| 17. VBM PCC left | .17 | .28* | .08 | –.37* | .33* | –.02 | –.32* | .48* | .04 | –.21 | .51* | –.01 | –.10 | .86* | .08 | –.34* |  |  |  |  |  |  |  |  |  |  |  |  |  |  |  |  |  |  |  |  |
| 18. MT PCC left | .13 | .23 | .75* | –.28* | .07 | .60* | –.18 | .13 | .72* | –.32* | –.04 | .68* | –.29* | .09 | .98* | –.34* | **.06** |  |  |  |  |  |  |  |  |  |  |  |  |  |  |  |  |  |  |  |
| 19. MD PCC left | –.01 | –.34* | –.26* | .28* | –.33* | –.19 | .33* | –.40* | –.52* | .71* | –.26* | –.44* | .67* | –.34* | –.41* | .87* | **–.33*** | **–.40*** |  |  |  |  |  |  |  |  |  |  |  |  |  |  |  |  |  |  |
| 20. VBM PCG right | –.02 | .26* | –.17 | –.09 | .25* | –.25* | –.08 | .26* | –.07 | –.11 | .34* | –.03 | –.16 | .43* | –.08 | –.33* | .33* | –.10 | –.21 |  |  |  |  |  |  |  |  |  |  |  |  |  |  |  |  |  |
| 21. MT PCG right | .15 | .35* | .68* | –.28* | .18 | .62* | –.20 | .05 | .78* | –.31* | .03 | .76* | –.22 | .05 | .78* | –.23 | –.05 | .78* | –.30* | **–.04** |  |  |  |  |  |  |  |  |  |  |  |  |  |  |  |  |
| 22. MD PCG right | .21 | –.12 | –.12 | –.11 | .03 | .03 | –.16 | –.13 | –.40* | .40* | –.14 | –.37* | .49* | –.14 | –.38* | .65* | –.08 | –.36* | .59* | **–.22** | **–.30*** |  |  |  |  |  |  |  |  |  |  |  |  |  |  |  |
| 23. VBM PCG left | .00 | .31* | –.22 | –.15 | .33* | –.29* | –.15 | .35* | –.10 | –.18 | .41* | –.06 | –.13 | .39* | –.10 | –.31* | .30* | –.13 | –.22 | .80* | –.08 | –.20 |  |  |  |  |  |  |  |  |  |  |  |  |  |  |
| 24. MT PCG left | .20 | .37* | .65* | –.31* | .20 | .64* | –.23 | .04 | .78* | –.33* | .03 | .79* | –.24 | .03 | .70* | –.21 | –.07 | .73* | –.30* | –.06 | .95* | –.25* | **–.11** |  |  |  |  |  |  |  |  |  |  |  |  |  |
| 25. MD PCG left | .25* | –.09 | –.08 | –.10 | .05 | .07 | –.15 | –.18 | –.43* | .44* | –.16 | –.41* | .52* | –.13 | –.35* | .67* | –.03 | –.32* | .61* | –.20 | –.29* | .95* | **–.21** | **–.27*** |  |  |  |  |  |  |  |  |  |  |  |  |
| 26. VBM JLC right | –.13 | .04 | –.11 | .09 | .09 | –.22 | .10 | .27* | .06 | –.08 | .36* | .05 | –.13 | .31* | .06 | –.35* | .28* | .02 | –.28* | .41* | .11 | –.38* | .40* | .04 | –.36* |  |  |  |  |  |  |  |  |  |  |  |
| 27. MT JLC right | .10 | .29* | .57* | –.28* | .12 | .50* | –.19 | .09 | .88* | –.42* | .04 | .84* | –.40* | .16 | .70* | –.39* | .04 | .70* | –.48* | –.04 | .83* | –.39* | –.15 | .86* | –.43* | **.09** |  |  |  |  |  |  |  |  |  |  |
| 28. MD JLC right | .21 | –.01 | –.05 | –.13 | .10 | .05 | –.15 | –.20 | –.34* | .43* | –.09 | –.32* | .46* | –.09 | –.27* | .64* | –.07 | –.24* | .60* | –.26* | –.21 | .81* | –.22 | –.17 | .83* | **–.37*** | **–.34*** |  |  |  |  |  |  |  |  |  |
| 29. VBM JLC left | –.17 | .18 | –.04 | –.08 | .21 | –.14 | –.08 | .33* | .11 | –.10 | .36* | .09 | –.09 | .43* | .12 | –.43* | .44* | .06 | –.34* | .42* | .09 | –.32* | .42* | .02 | –.29* | .80* | .08 | –.34* |  |  |  |  |  |  |  |  |
| 30. MT JLC left | .08 | .26* | .55* | –.24* | .09 | .47* | –.16 | .10 | .86* | –.37* | –.03 | .87* | –.36* | .04 | .74* | –.32* | –.04 | .76* | –.39* | –.07 | .85* | –.31* | –.16 | .89* | –.34* | .06 | .91* | –.28* | **.09** |  |  |  |  |  |  |  |
| 31. MD JLC left | .12 | .01 | –.08 | –.19 | .08 | .04 | –.18 | –.20 | –.42* | .41* | –.08 | –.39* | .46* | –.18 | –.37* | .66* | –.10 | –.35* | .62* | –.27* | –.28* | .80* | –.20 | –.23 | .85* | –.42* | –.43* | .83* | **–.42*** | **–.36*** |  |  |  |  |  |  |
| 32. VBM IFG right | .29* | .20 | –.22 | –.21 | .26* | –.21 | –.28* | .25* | –.02 | –.23* | .41* | –.04 | –.15 | .44* | –.14 | –.15 | .36* | –.14 | –.10 | .48* | –.07 | –.02 | .48* | –.07 | .01 | .21 | –.03 | .09 | .22 | –.07 | .00 |  |  |  |  |  |
| 33. MT IFG right | .22 | .32* | .68* | –.34* | .21 | .67* | –.27* | .04 | .70* | –.23 | .08 | .67* | –.17 | .07 | .72* | –.16 | –.03 | .71* | –.23 | –.10 | .87* | –.14 | –.13 | .82* | –.16 | .03 | .74* | –.08 | .03 | .72* | –.14 | **–.02** |  |  |  |  |
| 34. MD IFG right | –.05 | –.28* | –.08 | .37* | –.26* | –.04 | .35* | –.30* | –.40* | .46* | –.29* | –.35* | .50* | –.28* | –.23 | .35* | –.25* | –.23 | .47* | –.04 | –.26* | .41* | –.05 | –.25* | .41* | –.15 | –.40* | .26* | –.13 | –.33* | .30* | **–.37*** | **–.28*** |  |  |  |
| 35. VBM IFG left | .01 | .15 | –.20 | –.15 | .28* | –.08 | –.25* | .34* | –.27* | –.11 | .28* | –.33* | .00 | .38* | –.24* | –.14 | .39* | –.25* | –.13 | .33* | –.28* | .21 | .27* | –.24 | .20 | .08 | –.25* | .09 | .23* | –.23 | .10 | .47* | –.16 | –.09 |  |  |
| 36. MT IFG left | .26* | .32* | .55* | –.28* | .25* | .61* | –.27* | .01 | .71* | –.29* | .03 | .71* | –.22 | .01 | .46* | –.13 | –.11 | .48* | –.24* | –.07 | .81* | –.07 | –.16 | .89* | –.09 | –.02 | .74* | –.03 | –.05 | .74* | –.08 | –.01 | .76* | –.17 | **–.13** |  |
| 37. MD IFG left | .13 | –.17 | –.02 | .22 | –.14 | –.04 | .25* | –.21 | –.42* | .50* | –.22 | –.35* | .56* | –.18 | –.18 | .44* | –.06 | –.18 | .47* | .01 | –.18 | .45* | .07 | –.24 | .50* | –.03 | –.41* | .39* | –.05 | –.32* | .36* | –.14 | –.20 | .64* | **–.14** | **–.26*** |

*Note.* T1 = time point 1 (0 months), VO_2_peak = peak oxygen uptake, VBM = voxel-based morphometry, MT = magnetization transfer, MD = mean diffusivity, HC = hippocampus, ACC = anterior cingulate cortex, PCC = posterior cingulate cortex, PCG = precentral gyrus, JLC = juxtapositional lobule cortex, IFG = inferior frontal gyrus. Correlation coefficients across imaging modalities within regions of interest are in bold for visual clarity.

* *p* < .050
